# An Automated Customizable Live Web Crawler for Curation of Comparative Pharmacokinetic Data: An Intelligent Compilation of Research-Based Comprehensive Article Repository

**DOI:** 10.3390/pharmaceutics15051384

**Published:** 2023-04-30

**Authors:** Remya Ampadi Ramachandran, Lisa A. Tell, Sidharth Rai, Nuwan Indika Millagaha Gedara, Xuan Xu, Jim E. Riviere, Majid Jaberi-Douraki

**Affiliations:** 1 1DATA Consortium, Kansas State University Olathe, Olathe, KS 66061, USA; 2 Food Animal Residue Avoidance and Databank Program (FARAD), Kansas State University Olathe, Olathe, KS 66061, USA; 3 Department of Mathematics, Kansas State University, Manhattan, KS 66502, USA; 4 FARAD, Department of Medicine and Epidemiology, School of Veterinary Medicine, University of California-Davis, Davis, CA 95616, USA

**Keywords:** web crawler, digital object identifiers (DOIs), pharmacokinetics (PK), application programming interface (API), text and data mining (TDM)

## Abstract

Data curation has significant research implications irrespective of application areas. As most curated studies rely on databases for data extraction, the availability of data resources is extremely important. Taking a perspective from pharmacology, extracted data contribute to improved drug treatment outcomes and well-being but with some challenges. Considering available pharmacology literature, it is necessary to review articles and other scientific documents carefully. A typical method of accessing articles on journal websites is through long-established searches. In addition to being labor-intensive, this conventional approach often leads to incomplete-content downloads. This paper presents a new methodology with user-friendly models to accept search keywords according to the investigators’ research fields for metadata and full-text articles. To accomplish this, scientifically published records on the pharmacokinetics of drugs were extracted from several sources using our navigating tool called the Web Crawler for Pharmacokinetics (WCPK). The results of metadata extraction provided 74,867 publications for four drug classes. Full-text extractions performed with WCPK revealed that the system is highly competent, extracting over 97% of records. This model helps establish keyword-based article repositories, contributing to comprehensive databases for article curation projects. This paper also explains the procedures adopted to build the proposed customizable-live WCPK, from system design and development to deployment phases.

## 1. Introduction and Background

Pharmacokinetics (PK) is a subfield of pharmacology that models the time-concentration profile of drugs administered in vivo to living organisms (humans and animals) [1,2,3,4,5]. PK parameters including clearance, the area under the curve, volume of distribution, and other conditional descriptors such as dosage, route, and patient characteristics, all contribute to the effects of drug administration in humans and animals. Identifying, extracting, and analyzing such data from published records is undeniably beneficial in conducting PK studies [6]. There are also many clinical and research studies published in the literature discussing a wide range of drug PK analyses. Information retrieval in this field is challenging and time-consuming, as one must identify the desired publications, perform data scrapings, and download relevant articles, case studies, or clinical reports from the web URLs. Web crawling and web data extraction are the terms coined to represent such web browsing activities [7].

A text and data mining strategy (TDM), once automated, enables rapid and more efficient access to the enormous text and data resources available on the web through algorithms, services, or software tools such as web crawlers and spiders. As a result, TDM techniques provide researchers with a significant amount of up-to-date and comprehensive information on their topic of interest for analysis. Most scientific and scholarly publishers provide their own TDM application programming interfaces (APIs) to ease access to their scholarly content, provided that a valid license agreement is in place [8,9,10,11,12].

In combination with different TDM API service providers, such as Scopus, Springer, Crossref, arXiv, bioRxiv, medRxiv, BioMed Central, PubMed, Web of Science, IEEE, and Public Library of Science, a large number of scientific articles can be accessed [9,10,11,12,13,14,15,16,17,18,19,20]. The API Key can be obtained by researchers for free, and the APIs can be used for non-commercial purposes, subject to the policies of TDM API providers regarding API use and data sharing. API subscriptions may be required for any individual or organization using APIs for commercial purposes.

It is worth mentioning, however, that the most important challenge is the absence of a web crawler and scheduler that facilitates both article searching and extraction of relevant information. This is the rationale behind developing an automated web crawling paradigm that can function on a search query based on the topic of interest, in this case, the drug’s pharmacokinetic profiles, a crucial step in safety assessment and ultimate clinical application. The proposed web crawler will also directly contribute to the primary mission of the Food Animal Residue Avoidance and Depletion (FARAD) program by providing a platform capable of retrieving PK data across species. Through the establishment of an article repository for each drug class, this platform will enable producers, veterinarians, allied professionals, and researchers to have access to a TDM service at any time. The core function of this project is to maintain and provide a platform for organizing and extracting information from PK literature, which will have an interface to both structured and unstructured databases [21,22].

## 2. Methodology for PK Web Navigating/Crawling System

### 2.1. The Architecture of Web Crawler for PK (WCPK)

The focus of our research group is the importation and curation of published records to extract data from PK analysis in veterinary or human medicine, specifically to estimate the rate of depletion of drugs and chemicals in food-producing animals, as well as serving as a first step in developing a PK parameter web crawling model. Software architecture is developed to establish how system components of the navigating system are identified and cooperate, how they interact, and how the interface protocols for communication between them are defined. In a more formal sense, important items needed for the software architecture of this project are defined as follows:

- Identifying drugs with other names from the ATC Classification System and defining the same active ingredients for different routes of administration. Various sources and countries generate phrases and terminologies for each data field, and the drug names are represented by several aliases, including generic, brand (trade), and international names, medicinal formulations, active substances or active ingredients, as well as mismatched phrases and special (or international) characters. We then need to consolidate the combination of active ingredients, generic names, brand names, etc. Hence, the drug names are mapped to drug parents using the DrugBank database (Alberta Innovates–Health Solutions, The Metabolomics Innovation Center) [23] and the Kegg drug database [24,25]. These similar active ingredients are presented in Supplementary Materials Table S1;
- Metadata search queries from TDM API service providers such as Scopus, Springer, Crossref, arXiv, BioMed Central, PubMed, Web of Science, IEEE, and Public Library of Science must be defined. The following is a search query from the API service provider Scopus: Search ((ALL((drugs) AND (clearance) AND (volume of distribution) AND (route)))), for metadata retrieval;
- Full-text API calls: Scopus API, Springer API, Crossref REST API for full-text access;
- The automation of distribution servers and browsers using Selenium web drivers, which allows users to navigate online locally or remotely: Chrome driver for HTML and PDF downloads from journal websites [26,27];
- Automating the storage process in the 1DATA repository: Article repository for veterinary or human medicine classes obtained from the Anatomical Therapeutic Chemical (ATC) Classification System [28,29].

**Figure 1:**
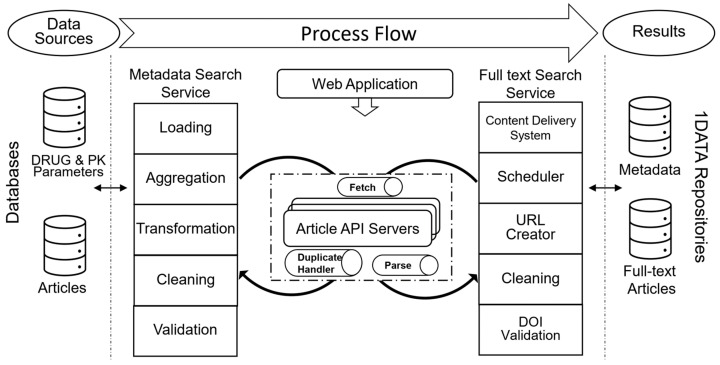
Overall architecture of the Web Crawler for PK analysis.

The overall architecture of the Web Crawler for PK (WCPK) analysis is depicted in Figure 1. As mentioned earlier, drug databases and article providers are the main data sources of WCPK. A metadata search service is invoked based on the drug names with corresponding PK parameters and generates a DOI collection. This DOI collection is given as input to the full-text search service. The outcome of the project is handled by the content delivery system where the files are stored in separate repositories of the 1DATA server: one for the metadata and another for the full-text downloads [30].

The proposed WCPK is built on a Python-based API wrapper to access Scopus, pybliometrics, and API providers from other publishers. The API wrapper written for the Scopus RESTful API assists in Scopus database searches and access through user-friendly interfaces [31]. This portion of the work provides the foundation for the rest of this research, in which we conduct and assess a database search based on PK parameters and the corresponding results that are performed to retrieve the available articles’ metadata and full-text records. The metadata and full-text retrieval part of the system play a critical role in consolidating the availability of articles and their basic information that meet the criteria for the search. It is evident that metadata scraping is a valuable tool for retrieving information about articles at a high level, where it provides significant insight into the content of the full article [32].

We are attempting primarily to retrieve full-text records from digital object identifiers (DOIs) [33]. We focus on detailed or end-to-end information extraction about the PK parameters and their reported results in this architecture development article. There are several types of full-text files that can be curated and imported for this purpose, including XML files, HTML files, JSON files, and PDF files. A Python 3.10 release is used for the development of this program module along with a variety of API options from Scopus, Springer Nature, and Crossref REST APIs [11,12,13,34]. The TDM APIs obtained are from the institutional subscription, with a weekly limit of 20,000 article downloads per week. API calls are used to facilitate services or information retrieval upon request, and, in our case, the request is a full-text URL together with the corresponding API key [35,36]. 

Moreover, the Crossref REST API for full-text article downloads includes an additional sequence of steps to determine the DOI registration agency (RA), followed by accessing the full-text link information. As a result of determining the RA as Crossref, unnecessary URL hits that are not supported by Crossref are avoided. The link information includes the type of files available and the full-text link that can be accessed for the given DOI [37]. Additionally, to Crossref, there are several other RAs, including CNKI (China National Knowledge Infrastructure), DataCite, ISTIC & Wanfang Data (The Institute of Scientific and Technical Information of China & Wanfang Data Co., Ltd., Beijing, China), mEDRA (Multilingual European DOI Registration Agency), and OP (Publications Office of the European Union). RAs are authorities recognized by the International DOI Foundation (IDF) to provide services including allocation of DOI name prefixes, registration of DOI names, and infrastructure to facilitate the declaration and maintenance of metadata and state data for the registrants [33,38,39]. 

**Figure 2:**
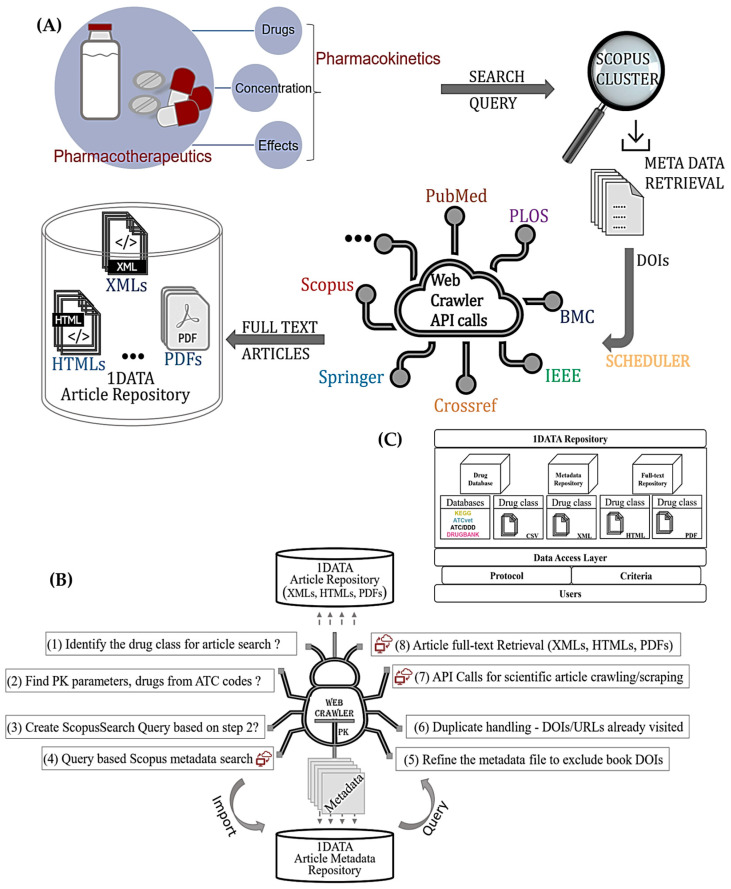
(**A**) Overall dataflow of PK Web Navigating/Crawling System with Scheduler. This shows how metadata and then full-text files are extracted in each step. (**B**) Overall workflow of Web Crawler with Scheduler for PK parameters data extraction. This flow diagram consists of multiple procedures from extracting metadata to full-text retrieval. (**C**) 1DATA repository model.

Figure 2A depicts the overall dataflow and Figure 2B depicts the workflow of the WCPK, consisting of three main modules dealing with DOI extraction, duplicate handling, and article retrieval through Scopus API, Springer API, and Crossref REST API open-access subscription content retrieval options. The full-text retrieval modules also offer a scheduling feature that facilitates a weekly instantiation of Scopus metadata search and full-text retrieval packages. In order to implement this periodic downloading option, Python’s in-process schedule library named ‘schedule’ is utilized, since it is a simple solution to scheduling problems [40,41,42]. The 1DATA repository in Figure 2C is enriched with multiple databases holding metadata, full-text articles, pharmacokinetic parameters, and so forth where users may be able to extract relevant information based on the drug classes [30].

### 2.2. The Module of Article Metadata Extraction

It was mentioned earlier that the module of Article Metadata Extraction is the basic building block of the WCPK. The focus of this module is on the interaction between our Python module and the Scopus database as one the most reliable indexing databases through the Python-based API wrapper pybliometrics and its *ScopusSearch()* function. As a result, we are able to access clusters of research articles, trends, and journals. Additionally, when compared with other article repositories, Scopus provides an article repository with a source-unbiased database containing millions of records, offers flexibility when designing queries, and allows the addition of filters, such as language, author, and topic selection based on user requirements [43,44]. Below, we provide the procedure for the pseudocode of a test query related to the PK parameters of interest in this study. For example, in this case, the search query consists of a combination of drug names, PK parameters such as clearance, the volume of distribution, and other PK descriptors such as routes of administration and species studied. A description of the procedure used to compile and extract metadata can be found in Procedure 1:
**Procedure 1. Module of Article Metadata Extraction****procedure** doi_scopusSearch (drug list, PK parameters):**begin** derive search query input variables from the procedure parameters based on drug class.  **if** variables# (drug names, clearance, volume of distribution, route) are available:  execute ScopusSearch query (input variables) to retrieve all metadata information.    **if** (outcome is NOT NULL): #       save metadata results to Data Frame      save metadata results to a CSV file.**end**output: articles metadata file for desired drug class

### 2.3. The Module of Duplicate Handling

The module of Duplicate Handling is designed to deal with the existing duplicate DOIs that are already present in the metadata file. Furthermore, this module provides filtering for book DOIs, Springer article DOIs, and the DOIs that have been visited and corresponding records extracted. DOIs that do not fall into these categories are considered for full-text access using Scopus API, due to its flexibility in offering a wide range of scientific articles independent of the journal or service providers. Certain URL access may be avoided, for instance, irrelevant API calls or previously accessed URLs. This is key for the performance aspect of this developed model. This is achieved by looking up the data captured in the reference file that has the URLs visited with details on the DOIs prior to making any of the functional calls, see Procedure 2 below for more information.
**Procedure 2. Module of Duplicate Handling****procedure** doi_duplicateHandling (doi, subtype, subtypeDescription):**begin** input variables—doi, subtype, subtypeDescription from the metadata file **if** input variables are NOT NULL:  invoke duplicateHandler module:  **if** doi is NOT NULL:   drop duplicates if exist. **if** (subtype is bk): save doi with corresponding information in book-doi CSV file. **else:** invoke springerLookup module:   **if** doi matches certain semantic rules:     save doi information in springer-doi CSV file.   **else**:     save doi information in Scopus-doi CSV file.**end**output: book-doi, Springer-doi, Scopus-doi CSV files.

### 2.4. The Module of Full-Text Retrieval

In this module, the DOI groups from the Duplicate Handling module are being sent to the desired API classes. As explained earlier, even for full-text article retrieval, a weightage is given to the Scopus API search using its flexibility to offer a source-neutral heterogeneous scientific article repository. Candidate records for full-text retrieval are typically preferred in the form of XMLs, JSONs, HTMLs, and PDFs in decreasing order of importance. When a particular API call returns a NULL sequence, the DOI is categorized and recorded as an unsuccessful hit category and handled separately, see Procedure 3 below for more information.
**Procedure 3. Module of Full-Text Retrieval****procedure** doi_fulltextRetrieval (doi, API key, file-folder):**begin**input variables—doi, API key, file-folder**for** doi in doi-list:   **case 1:**      append doi to doi_visited list.      invoke **Scopus-API** handler:      **if** webpage returns:          full text (XML, or JSON) then          save content.     **elseif:** full text(HTML) then                     **if** the Methods and Results sections return true then             save content.     **else:**         append doi to unsuccessful.csv file.    **case 2:**      append doi to doi_visited list      invoke **Springer-API** handler:      **if** webpage returns:          full text (XML) then         save content.     **else:**         append doi to unsuccessful.csv file.   **case 3:**      append doi to doi_visited list.      invoke **Crossref-API** handler:      **if** webpage returns:          full text (XML, or PDFs) then         save content.     **else:**         append doi to unsuccessful.csv file.   **case 4:**      unsuccessful doi list      append doi to doi_visited list.      **if** a PDF link is available then          save the content.     **else:**         return incomplete-DOI list.   **end**output: XMLs, HTMLs, PDFs, incomplete-DOI list

In this way, scientific article repositories such as Scopus, Springer, and Crossref are mainly being integrated into the WCPK for full-text downloads. However, the initial phase of metadata retrieval deals with only the Scopus database, considering its advantages over other API calls such as a source-neutral, heterogeneous collection of metadata and article storage.

## 3. Results

The WCPK search engine deployed on Python 3.10 focused on drug classes obtained from the ATC Classification System [28,29]. Published records were extracted from various sources on the pharmacokinetics aspects of Anti-infectives for Systemic Use (ATC code QJ), Dermatologicals (ATC code QD), Antiparasitic Products, Insecticides, and Repellents (ATC Code QP), steroid anabolic growth promoters from Systemic Hormonal Preparations Excluding Sex Hormones and Insulin (ATC Code QH), and some other drugs such as ionophores (See Table 1) [45]. It is important to point out that this model can be applied to any custom-built dataset constructed according to the DOI standard. With the Python-based API wrapper Pybliometrics package for the metadata extraction [31], a search query (SQ) was developed, as shown below marked as SQ (1). 

SQ (1): ALL (“Drugs” AND (“cl” OR “clearance” OR “cleared”) AND (“distribution volume” OR “volume of distribution”) AND “Routes”); where the drugs and routes are imported into the search query from the following list (Table 2) for a given drug class.

In this study, we searched for all PK terms to be present when a query was given by the operator “AND” for drugs, drug clearance, the volume of distribution, and route combinations, while, within each category from a particular PK parameter, the focus was given to the availability of at least one parameter to be True (the operator “OR”). Drugs used in the search query SQ (1) for each drug class are provided in Supplementary Materials Table S1.

**Figure 3:**
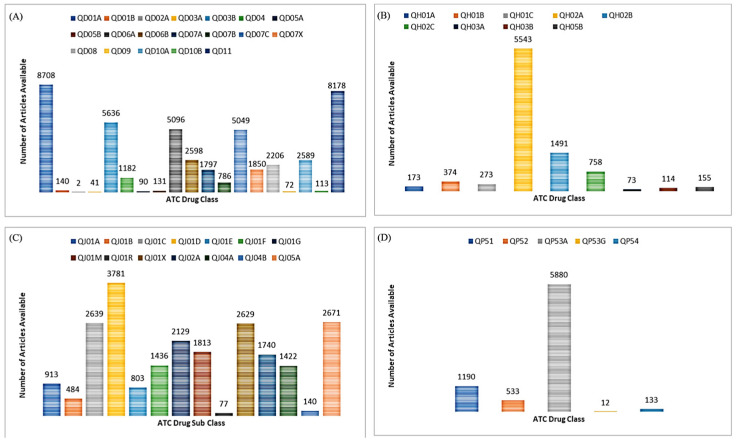
Metadata analysis for the number of articles available for the drug class (**A**) QD01–QD11 (Dermatologicals), (**B**) QH01-QH05 (Systemic Hormonal Preparations Excluding Sex Hormones and Insulin), (**C**) QJ01–QJ05 (Antiinfectives for Systemic Use), (**D**) QP51–QP54 (Antiparasitic Products, Insecticides, and Repellents) using the Scopus search query in SQ (1).

The search query SQ (1) refers to the ATC drug class QJ01 classification for Antibacterials for Systemic Use, and the metadata outcome provides various information including fields of data such as DOI, title, abstract, authors, affiliations, etc. The number of articles logged for the QD01–QD11, QH01–QH05, QJ01–QJ05, and QP51–QP54 drug classes categorical search queries shown in Figure 3 confirm the vast collection of literature that exists in the field of pharmacology. It should be noted that the metadata file itself can be a viable candidate for article information scraping. This is because it can provide a more general understanding of the article’s subject matter. A representative outcome of metadata retrieval for search query SQ (1) is provided in Table 3. 

The metadata information retrieved for other article types including review articles in this study, and book chapters and books are shown in Supplementary Materials Table S2. Apart from the metadata file, this step also gives insights into the number of scientific articles available for each drug class, as depicted in Figure 3. This figure provides the number of articles available in the literature for anti-infectives for systemic use, QJ (*n* = 22,677), dermatologicals, QD (*n* = 35,488), systemic hormonal preparation, excl. sex hormones and insulin, QH (*n* = 8954), and antiparasitic products, QP (*n* = 7748). It is worth noting that multiple occurrences of the same DOIs are likely to be included in two or more drug classes. Our search query also provides further information on the number of articles available at each level of the ATC code. For instance, the first level QJ drug class indicating the anatomical main group with its therapeutic subgroup (the second level QJ01) shows the presence of 22,677 articles, while the therapeutic/pharmacological subgroup has the following numbers of available articles: J01A: 913, J01B: 484, J01C: 2639, J01D: 3781, J01E: 803, J01F: 1436, J01G: 2129, J01M: 1813, J01R: 77, J01X: 2629, J02A: 1740, J04A: 1422, J04B: 140, and J05A: 2671. It is possible to investigate further other article properties such as chemical, therapeutic, or pharmacological subgroups (level four), and chemical substances (level five). This study has been limited to ATC level three [28,29].

**Figure 4:**
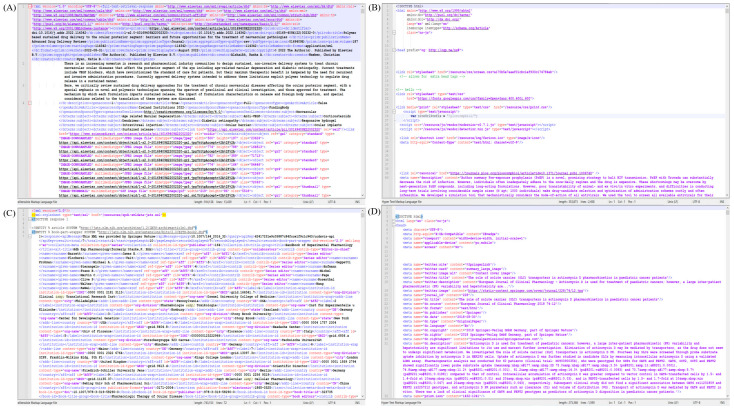
Full-text articles retrieved from Scopus API (**A**) XML, (**B**) HTML, and Springer API, (**C**) XML, and (**D**) HTML file types. Only a portion of the extracted files are shown here.

Since our proposed WCPK is solely designed to handle article DOI, subtype, and subtypeDescription data from the metadata search query output, these data will then be sent to the Duplicate Handling module. Pandas data frame has been utilized to limit the data selection to DOI, subtype, and subtypeDescription [46]. Metadata information, as such, is being handled by our text analytics team for advanced exploratory data analysis [47]. The Duplicate Handling module then returns three different DOI groups including Scopus APIs, Springer APIs, and book categories. It is then followed by the module of Full-Text Retrieval to download the article in the given formats. For instance, with the Scopus dataset, the Scopus DOI handler is invoked and performs an API call and search for the availability of XML or HTML full-text content. Upon successful completion of the search, either the XML or HTML file is saved in the 1DATA article repository or elsewhere. Unsuccessful hits are marked, and the corresponding DOI is saved in a separate CSV file by appending the DOI to the incomplete file list. The Springer DOI handler is invoked and runs simultaneously for the Springer dataset in the same sequence as the Scopus handler for the Scopus dataset. Figure 4 displays a portion of the full-text articles (XMLs and HTMLs) generated by the Scopus API and Springer API, respectively.

Figure 4 shows different formats of file retrieval for the portion of XML files (Figure 4A,C) and HTML files (Figure 4B,D) from Scopus and Springer APIs. From an article curation perspective, the XML and HTML file tags are important due to their routine usage for data extraction based on need. Retrieving full-text files from each of the APIs separately might avoid any chances of data loss due to data fetching based on incompatible tags. 

Since the dataset given to the Scopus DOI handling module has all the DOIs except Springer DOIs and book DOIs, some of the URL hits are incomplete or unsuccessful in terms of full-text article retrieval. As a result of such occurrences, we have implemented the Crossref TDM API Click with the help of a service provided along with ORCID [37,48].

Using the Crossref TDM service, we first verified that the given DOI belongs to the Crossref agent and then, as shown below, retrieved the detailed information. From the examples of agency check outcomes listed below (Table 4), we can see mEDRA and ISTIC as article providers other than the Crossref [38,39]. It is worth noting that Table 4 is a more refined version of the outcomes while the actual encoded results obtained are given in Supplementary Materials Table S3. In this manner, we can filter out the full-text DOIs accessible with the Crossref provider, thus avoiding unauthorized or unnecessary URL hits.

**Figure 5:**
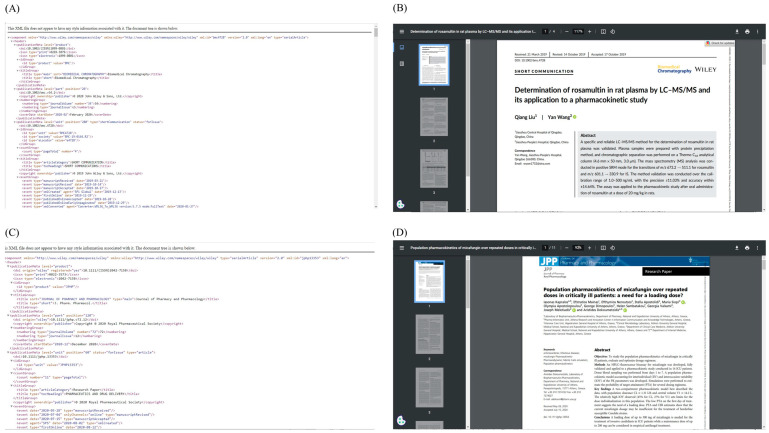
A portion of the full-text articles for the doi 10.1002/bmc.4728 [49] (**A**) XML [49], and (**B**) PDF [49], and doi 10.1111/jphp.13353 [50] (**C**) XML [50], and (**D**) PDF [50] file types retrieved from Crossref APIs are shown here.

In addition to identifying the DOIs provided by Crossref, it is needed to examine the information service package called *info()* provided by Crossref to identify the full-text options or application types such as XML, JSON, HTML, or PDF [37]. For example, we include a snippet below that shows the *info()* service obtained for the DOI 10.1002/bmc.4728 in Table 5 [49]. Actual encoded outcomes obtained for the information given in Table 5 are provided in Supplementary Materials Table S4. If the full-text option is available for the selected DOI, the *info()* service returns full-text links followed by the filetype variable (e.g., PDF or XML), in addition to the author details and TDM license information. Based on our interest in the file type for article data curation, the desired full-text link can be taken for further steps. Using Crossref API, snapshots of the portion of full text retrieved for the above-mentioned DOI 10.1002/bmc.4728 (A) XML [49], (B) PDF [49], and for the DOI 10.1111/jphp.13353 (C) XML [50], and (D) PDF [50] types are shown in Figure 5.

The remaining DOIs, which are stored in the CSV file called the unsuccessful or incomplete list, are handled separately to download the HTML or PDF file types using a different API called the Selenium web driver with its Chrome driver option facilitated through Python [26]. Our interest in extracting full text as a PDF file type is minimal. Therefore, this module is treated as a last resort. All the file formats, i.e., XMLs, HTMLs, JSONs, or PDFs, are accessed through the DOI system [33] link appended with its unique DOI obtained from the metadata. The purpose of this is to avoid visiting web pages that are unwanted or unauthorized when downloading scientific articles.

**Figure 6:**
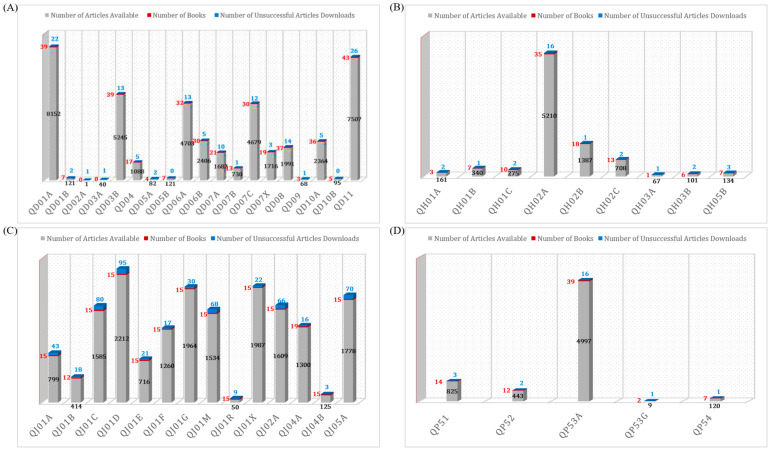
Drug category with the number of full-text article downloads (**A**) QD01-QD11 (Dermatologicals), (**B**) QH01-QH05 (Systemic Hormonal Preparations Excluding Sex Hormones and Insulin), (**C**) QJ01–QJ05 (Antiinfectives for Systemic Use), and (**D**) QP51-QP54 (Antiparasitic Products, Insecticides, and Repellents) through various article interfaces including Scopus API, Springer API, and Crossref APIs.

A graph Is presented in Figure 6 that shows an estimate of how many full-text articles were downloaded for the QD01–QD11 (Dermatologicals), QH01-QH05 (Systemic Hormonal Preparations Excluding Sex Hormones and Insulin), QJ01–QJ05 (Antiinfectives for Systemic Use), and QP51–QP54 (Antiparasitic Products, Insecticides, and Repellents) drug classes case study set. It is evident that, except for the book DOIs (red-colored box plots), we have downloaded the full text of almost every DOI. As a result of the downloaded articles, we were able to create a QD, QH, QJ, and QP reusable article repository, which is useful for the categorical analysis of data as well as for its blend analysis. Figure 6 also shows the number of unsuccessful article downloads, especially from the publishers such as Future Medicine, Future Science, Dustri-Verlag, Bentham Science, Oxford Academic–Transactions, and Pharmacological Reports, for which we do not have an active subscription [54,55,56,57,58,59]. Together with book DOIs, unsuccessful article DOIs will be handled separately for the final version of the proposed WCPK tool.

## 4. Discussion

When analyzing pharmacological data, it is often necessary to consider the properties, dynamics, and effects of a drug dose once it has been administered to a living organism [60,61,62]. As a result, an increasing number of data mining studies are being conducted in pharmacology to understand the properties and effects of a specific class of drugs [63,64]. These show the impact of these mining paradigms in healthcare applications where text or data mining provides useful information [62,65,66,67]. These studies are found in scientific literature, social media resources, and pharmacovigilance sources, for instance, provided by the US Food and Drug Administration (FDA) [47,68,69,70]. In a sense, these studies contribute to the establishment of an evidence-based drug safety protocol. Apart from the scientific literature, researchers also rely on international databases for data curation, analysis, and other research purposes. One of our recent studies falls into this category where we curated multiple databases to extract global maximum residue limits (MRLs) data for developing an automated MRL prediction model [71]. 

Irrespective of the application area, the interaction, and trends of scientific knowledge, as well as determining parameters and guidelines for statistical analysis, are influenced by metrics such as scientometrics, bibliometrics, and informetrics [72]. Open Knowledge Maps is one such bibliometric tool that facilitates a visual interface of the scientific data for the topic of interest, where a topical overview of the topics and their relatedness will be comprehensible [73]. CiteseerX, one of the pioneer digital library search engines, supports academic communities with a collection of all open-access articles under one roof. It also provides metadata information from the full text through both automatic and manual procedures [74]. The route of such information retrieval is through different crawler algorithms or tools such as Web of Science and Google Scholar [75] or scraping online web pages [76]. The success of webometrics studies present in the literature also relies on the effectiveness of search engines or crawlers adopted for information retrieval [77,78,79]. Similarly, almost all bibliometric data mining studies reported in the literature use scientific articles, case reports, or reviews as the primary source of information. Despite this apparent depth of data, there is a gap in the development of a systematic webpage navigator/crawler capable of automatically downloading articles of interest. The absence of a systematic web crawler that can function based on a drug and its associated PK parameter search query is a significant limitation since PK parameters are often the first step in evaluating drug safety and efficacy. Since data mining is an inevitable part of pharmacology and pharmacotherapeutics research [60], the creation of an article repository that can provide articles of interest based on the drug effects or its PK parameters would be of great benefit and assistance to researchers. 

Through the mining of web crawler algorithms, various possibilities are verified, including breadth-first (search the neighbors at the same level), depth-first (traverse to the bottom from the root node), URL ordering (queue), page-rank (importance based on the number of backlinks or citations), online page importance (importance of a page in a website), largest sites first (websites with the largest number of pages), page request—HTTP or the dynamic, customized site map (applicable to deal with updates on already visited pages), and filtering (query-based approach) [7,80,81]. In some of these algorithms, keywords are accepted as the search query, and all relevant URLs fulfilling that search query are returned. The next step is to select a URL that meets our needs or interests based on this information. This also indicates the limitations of existing crawling methods where we need to identify the URL and initiate the crawler to obtain information. It may not be feasible when it comes to the analysis of big data. A closer look at each of these methods reveals that, in most cases, human intervention is required to download appropriate articles or webpages where an automated crawler algorithm that functions based on certain criteria is found missing. 

The number of records in Table 6 represents the results of a basic/advanced search of the respective article repositories for the search queries SQ2, SQ3, and SQ4, regardless of the subscription criteria. Compared with SQ1, these are simple search queries, and the query turn-around time was faster for most crawlers. Apart from Scopus and WCPK, other repository searches failed for SQ1, with warnings such as ‘try a shorter query’, ‘try keywords instead of long phrases’, or ‘too long query’. As opposed to being a source-neutral repository for scientific articles such as Scopus, Crossref, and so forth, our proposed WCPK is capable of handling long queries for metadata, and thus retrieval of articles. An added advantage of WCPK is that it automatically handles the full-text retrieval module from metadata search results. With WCPK, we plan to integrate most article providers into a single framework to facilitate a unified solution for data mining studies in the field of pharmacokinetics. It can later be enhanced to incorporate most of the article service providers listed here in Table 6.

In addition, based on our understanding, most of the web crawlers discussed here are created from AI training models and extract information based on the metadata content. In the majority of cases, full-text information extraction seems to be manual. The curated data are also retrieved exclusively from open-access articles. Here comes the importance of our crawler, where we rely on institutional-based API keys to receive metadata and full-text content. As a result, we have the opportunity to deal with significantly higher records irrespective of their proprietary nature, copyrighted or open access, for data curation and analysis studies. The method that we adopted to implement our proposed crawler for PK analysis provides a novel approach to web crawling.

A biomedical web crawler currently in use can predict the importance of a website based on the topic of the website it addresses [82]. Other web crawlers from the PubMed search follow by URL identification and article download using the GNU Wget web crawler [83,84], as well as online visibility assessment of Biomedical Text Mining (BioTM) tools and resources with JSOUP java API [85,86]. However, crawlers are reported to focus on downloading structured data for data mining and analysis, which involves the data crawling, curating, or scraping of web or article content, including e-health applications [87]. An application of the distributed crawler framework ‘Scrapy-Redis-Bloomfilter’ is presented by Zhang et al. in [88] for analyses of the effect of change in outbreaks in public feeling. BioPatentMiner is another model deployed to retrieve information from patents that pertain to biomedical ontologies [89].

Furthermore, the proposed WCPK has been developed to fill the gap of a web crawler capable of handling full-text downloads according to the topic of interest from Scopus, Springer, or Crossref article providers. For this, researchers should develop a metadata search query for their topic of interest and extract the metadata information from the Scopus database through the metadata search service. Upon submitting the metadata file to the full-text search service, APIs will be invoked corresponding to DOIs, and full-text articles will be automatically downloaded. Incomplete DOI hits will be recorded for alternative consideration.

Among the main advantages of this model are (a) permits incorporation of a SCOPUS search query to assess the article availability, (b) functions on the TDM APIs of scientific article providers, (c) performs a duplicate check before hitting the URL, and (d) keeps track of incomplete URLs and unsuccessful attempts. This model also holds task scheduling possibilities for the metadata retrieval and initial full-text downloading phase, where SCOPUS and Springer API calls come into the picture. It is made possible with the help of DOI categorization implementing regular expression (regex) [90,91,92] for the DOIs from different article providers/agents. Another novelty of this approach, when compared with other crawling algorithms, is in the file formats of full-text articles being downloaded. Here, we are focused on XMLs, JSONs, HTMLs, and PDFs in order of importance.

As a case study, we have shown drug classes from the ATC classification [28,29] with PK parameters clearance, the volume of distribution, and other associated PK parameters including Route. Various classes of drug pharmacokinetics were collected including drug classes from Anti-infectives for Systemic Use (ATC code QJ), Dermatologicals (ATC code QD), Antiparasitic Products, Insecticides, and Repellents (ATC Code QP), steroid anabolic growth promoters from Systemic Hormonal Preparations Excluding Sex Hormones and Insulin (ATC Code QH), and some other drugs such as ionophores. The number of articles with metadata retrieved for each of the drug classes and subclasses is represented in Figure 3. A significant advantage of this model is that it allows almost complete coverage of the articles available online, by means of retrieving SCOPUS metadata as an initial step. As indicated by the search query, it is evident that some duplicate DOIs exist in the metadata information; however, they are handled by the duplicate handler module. This approach has the additional advantage of being flexible enough to accommodate any number or type of keywords, depending on the research interest or study area. Moreover, we have implemented TDM API calls for each article provider or agency to minimize or eliminate the possibility of unauthorized URL hits. All these factors and aspects contribute to the deployment of a high-performance, time-efficient WCPK.

Additionally, the proposed web crawler contains several other significant advantages, including the ability to access a large number of available articles, as well as the ability to extract metadata. The combination of full-text results indeed contains duplicate information, yet it offers the possibility of working with the curation of individual drug classes and generating structured data from the vast number of resources available on the internet. This feature also provides users with the ability to track incomplete article downloads that may need to be handled manually. Consequently, this model ensures significant improvements in the process of article extraction for scientific curation studies, and keeping the articles in the article repository with corresponding drug labels facilitates ease of article access later. 

Specifically, the proposed model will be able to contribute to the FARAD mission [93], a university-based national program funded for over 40 years by the United States Department of Agriculture (USDA) to ensure human food safety by ensuring that drug and chemical residues are not present in the edible products of food-producing animals (e.g., meat, milk, and eggs). This model will be deployed as an initial module of the automatic curation of scientific article projects to extract animal drugs, drug concentration, and many other associated PK parameters that are useful in predicting the depletion of drug and chemical residues in food animals.

In a way, the proposed WCPK supports our 1DATA initiative where we envision a common platform for researchers and other academic or industrial partners to collaborate and benefit from a unified resource of pharmacokinetic data. This can later be enhanced in other areas of pharmacology and biomedical applications. The 1DATA repository model that we are enriching has a variety of data repositories holding drug data associated with pharmacokinetics parameters, adverse effects, gene associations, and so on. Another feature that we are focusing on is the collection of scientific articles, case studies, and reports, which are labeled based on ATC drug categories or disease models. These full-text articles can then be easily curated for table, image, and text data mining studies. A consolidated database from the curated models will also be added to the 1DATA repository in the future [30].

Some of the challenges that we faced in the early phase of the WCPK implementation were with the full-text HTML downloading component, where the incomplete HTML contents were handled by incorporating an additional check in the program module for the presence of sections such as methods and results or discussion in each manuscript. This was done to override the HTML content downloading, which was limited to the abstract and/or summary sections. Another challenge was related to saving XML content as an HTML file type. This often happened when we tried to access already retrieved DOI content. This issue was resolved by incorporating the module of Duplicate Handling so that any duplicate attempt for full-text retrieval is avoided. Since we have a separate list for book DOIs and unsuccessful article download DOIs, we are formulating innovative approaches to handle these scenarios, which consist of around one percent of books and less than one percent of unsuccessful DOIs of published records. This part will be considered for a future more advanced implementation of WCPK. It is also worth mentioning that, for the smooth running of this web crawler, the institution/library must obtain TDM API keys, which can be considered a prerequisite for the successful deployment of the proposed model. Future development of this model may include (a) deploying a sophisticated scheduling algorithm for more effective resource and time utilization, (b) improving article repository storage rules and criteria, (c) deploying a web-based interface for WCPK starting from search query initiation, (d) deploying a gateway service for article curation projects, and (e) enhancing the model to handle other research-based search queries and web crawling. 

We also envision a live web crawler where users can select the research areas, keywords for the search query, full-text file formats, and curated data from both metadata and full-text articles, based on their field interests for further analysis, while keeping the proprietary nature of all the records. We assume that readers always have access to the globally accepted current formats such as XML, HTML, and PDF. In our WCPK analysis, for each API call, we have created separate modules handling different API responses. If, in the future, a new format is provided by the API service providers, the live web crawler may need to be edited for the corresponding modules to address any new response format changes (other than XML, HTML, or PDF). As part of the future plan, we aim to identify the available formats and to automatically take necessary steps as a result of potential challenges related to format changes or metadata, or full-text retrieval modifications in API services.

## 5. Conclusions

The customized WCPK proposed in this paper can support researchers to design bibliometric studies [47]. This model blends different bibliography providers in a single line by incorporating agent-specific TDM API calls to download full-text scientific articles, case reports, and book chapters. By addressing the presence of duplicate DOIs, visited DOIs, and incomplete web pages, the model guarantees a scheduled high-performance time-efficient platform for the automatic downloading of articles based on the research interests and needs.

## Figures and Tables

**Table 1:** The following drug classes were employed for full-text retrieval using the Anatomical Therapeutic Chemical (ATC) Classification System [28,29].

QJ	QD	QP	QH
QJ01 Antibacterials for Systemic Use QJ02 Antimycotics for Systemic Use QJ04 Antimycobacterials QJ05 Antivirals for Systemic Use QJ51 Antibacterials for Intramammary Use QJ54 Antimycobacterials for Intramammary Use	QD01 Antifungals for Dermatological Use QD02 Emollients and Protectives QD03 Preparations for Treatment of Wounds and Ulcers QD04 Antipruritics, Incl. Antihistamines, Anesthetics, Etc. QD05 Drugs for Keratoseborrheic Disorders (Atc Human: Antipsoriatics) QD06 Antibiotics and Chemotherapeutics for Dermatological Use QD07 Corticosteroids, Dermatological Preparations QD08 Antiseptics and Disinfectants QD09 Medicated Dressings QD10 Anti-Acne Preparations QD11 Other Dermatological Preparations QD51 Products for the Treatment of Claws and Hoofs	QP51 Antiprotozoals QP52 Anthelmintics QP53 Ectoparaciticides, Insecticides, and Repellents QP54 Endectocides	QH01 Pituitary and Hypothalamic Hormones and Analogues QH02 Corticosteroids for Systemic Use QH03 Thyroid Therapy QH04 Pancreatic Hormones QH05 Calcium Homeostasis

**Table 2:** This table was used for the drugs and routes imported into the search query in SQ (1) for a given drug class.

Parameter	Items in Each Query Sent via Scopus Search in SQ (1)
Drugs	“arestin” OR “aureomycin” OR “bristacycline” OR “chlortetracycline” OR “chlortetracycline AND and AND bisulfate” OR “chlortetracycline AND and AND hydrochloride” OR “clomocycline” OR “declomycin” OR “demeclocycline” OR “demeclocycline AND and AND hydrochloride” OR “demethylchlortetracycline” OR “demethylchlortetracycline AND and AND hydrochloride” OR “doxychel” OR “doxycycline” OR “doxycycline AND and AND calcium” OR “doxycycline AND and AND fosfatex” OR “doxycycline AND and AND hyclate” OR “doxycycline AND and AND hydrate” OR “doxycycline AND and AND hydrochloride” OR “doxycycline AND and AND hydrochloride AND and AND hydrate” OR “dynacin” OR “eravacycline” OR “eravacycline AND and AND dihydrochloride” OR “lymecycline” OR “lymepak” OR “metacycline” OR “methacycline” OR “methacycline AND and AND hydrochloride” OR “minocin” OR “minocycline” OR “minocycline AND and AND hydrochloride” OR “monodox” OR “nuzyra” OR “omadacycline” OR “omadacycline AND and AND tosylate” OR “oracea” OR “oxytetracycline” OR “oxytetracycline AND and AND calcium” OR “oxytetracycline AND and AND dihydrate” OR “oxytetracycline AND and AND hydrochloride” OR “penimepicycline” OR “periostat” OR “rolitetracycline” OR “rolitetracycline AND and AND nitrate” OR “rondomycin” OR “sarecycline” OR “sarecycline AND and AND hydrochloride” OR “seysara” OR “solodyn” OR “sumycin” OR “synterin” OR “terramycin” OR “tetracycline” OR “tetracycline AND and AND hydrochloride” OR “tetracycline AND and AND hydrochloride AND and AND epidihydrocholesterin” OR “tetracycline AND and AND hydrochloride AND and AND hydrocortisone AND and AND acetate” OR “tetracycline AND and AND metaphosphate” OR “tetracycline AND and AND phosphate AND and AND complex” OR “tetracycline AND and AND presteron” OR “tetrax” OR “tigecycline” OR “tygacil” OR “vibramycin” OR “xerava”
Routes	“i.m” OR “i.m. OR “im” OR “im” OR “Intra” OR “intraarterial” OR “intra-arterial” OR “intra-articular” OR “intra-articularly” OR “Intramammary” OR “Intramuscular” OR “Intra-muscular” OR “Intranasal” OR “intravenous” OR “nasogastric” OR “ocular” OR “ointment” OR “oral” OR “orally” OR “OTM” OR “p.” OR “p.o” OR “p o.” OR “p.o.” OR “po” OR “s.c” OR “s.c.” OR “sc” OR “sc.” OR “Subcutaneous” OR “Topically” OR “trans” OR “transdermal” OR “trans-dermal” OR “transdermally” OR “transmucosal” OR “dermal” OR “nasal” OR “o” OR “Topical”

**Table 3:** Metadata information collected for an original article type of the drug class QJ01D from the Scopus search query is taken as a representative outcome.

Extracted Data from Scopus Metadata	*eid|doi|pii|pubmed_id|title|subtype|subtypeDescription|creator|afid|affilname|affiliation_city|affiliation_country|author_count|author_names|author_ids|author_afids|coverDate|coverDisplayDate|publicationName|issn|source_id|eIssn|aggregationType|volume|issueIdentifier|article_number|pageRange|description|authkeywords|citedby_count|openaccess|freetoread|freetoreadLabel|fund_acr|fund_no|fund_sponsor*
Example of metadata obtained from Scopus	2-s2.0-85131400463|10.1016/j.ejps.2022.106219|S092809872200104X|35,618,200|Amikacin pharmacokinetics in elderly patients with severe infections|ar|Article|Medellín-Garibay S.E.|60032541; 60031335; 60025844; 60,016,574|Hospital Severo Ochoa; Universidad Autonoma de San Luis Potosi; Hospital Universitario Puerta de Hierro Majadahonda; Universidad Complutense de Madrid, Facultad de Farmacia|Leganes;San Luis Potosí;Majadahonda; Madrid|Spain; Mexico; Spain; Spain|8|Medellín-Garibay, Susanna E.; Romano-Aguilar, Melissa; Parada, Alejandro; Suárez, David; Romano-Moreno, Silvia;Barcia, Emilia;Cervero, Miguel;García, Benito|36610623800; 57210937049; 57729583200; 57730179100; 57218390495; 6603720469; 57222997862; 49761285500|60031335; 60031335; 60031335; 60032541-60025844; 60031335; 60016574; 60032541-60025844; 60032541-60025844|8/1/2022|1-Aug-2022|European Journal of Pharmaceutical Sciences|9,280,987|21,331|18,790,720|Journal|175||106219| |Objective: The aim of this study was to characterize the population pharmacokinetics of amikacin in elderly patients by means of nonlinear mixed effects modelling and to propose initial dosing schemes to optimize therapy based on PK/PD targets. Method: A total of 137 elderly patients from 65 to 94 years receiving intravenous amikacin and routine therapeutic drug monitoring at Hospital Universitario Severo Ochoa were included. Concentration–time data and clinical information were retrospectively collected; initial doses of amikacin ranged from 5.7 to 22.5 mg/kg/day and each patient provided between 1 and 10 samples. Results: Amikacin pharmacokinetics were best described by a two-compartment open model; creatinine clearance (CrCL) was related to drug clearance (2.75 L/h/80 mL/min) and it was augmented 28% when non-steroidal anti-inflammatory drugs were concomitantly administered. Body mass index (BMI) influenced the central volume of distribution (17.4 L/25 kg/m^2^). Relative absolute prediction error was reduced from 33.2% (base model) to 17.9% (final model) when predictive performance was evaluated with a different group of elderly patients. A nomogram for initial amikacin dosage was developed and evaluated based on stochastic simulations considering final model to achieve PK/PD targets (Cmax/MIC > 10 and AUC/MIC > 75) and to avoid toxic threshold (Cmin < 2.5 mg/L). Conclusion: Initial dosing approach for amikacin was designed for elderly patients based on nonlinear mixed effects modeling to maximize the probability to attain efficacy and safety targets considering individual BMI and CrCL.| Antiinfectives, Clinical pharmacokinetics, Individualized drug therapy, Pharmacometrics, Population pharmacokinetics, Special populations, Therapeutic drug monitoring|0|1|publisherfullgold|Gold||undefined| |

**Table 4:** DOI registration agency checks to confirm whether the DOI is associated with the service provided by Crossref APIs.

Query URL	Outcome
https://api.crossref.org/works/10.12998/wjcc.v10.i18.6218/agency (accesses on 25 October 2022)	status: ok message-type: work-agency message-version: 1.0.0 message: {DOI:10.12998/wjcc.v10.i18.6218} agency: { id: crossref, label: Crossref}
https://api.crossref.org/works/10.22038/ijp.2017.26942.2320/agency (accesses on 25 October 2022)	status: ok message-type: work-agency message-version: 1.0.0 message: {DOI:10.22038/ijp.2017.26942.2320} agency: {id: medra, label: mEDRA}
https://api.crossref.org/works/10.3760/cma.j.issn.2095-4352.2019.11.001/agency (accesses on 25 October 2022)	status: ok message-type: work-agency message-version: 1.0.0 message: {DOI: 10.3760/cma.j.issn.2095-4352.2019.11.001} agency: {id: istic, label: ISTIC}

**Table 5:** Crossref information retrieval for some of the selected DOIs 10.1002/bmc.4728 [49], 10.1111/jvp.13054 [51], 10.1111/jphp.13353 [50], 10.1111/jphp.12275 [52], and 10.1211/jpp.59.8.0004 [53] to obtain the full-text (XML, PDF) links.

Python Code Snippet	Outcome
r = opener.open(‘http://dx.doi.org/10.1002/bmc.4728’) print (r.info()[‘Link’])	https://onlinelibrary.wiley.com/doi/pdf/10.1002/bmc.4728, type=“application/pdf” (accessed on 28 October 2022) https://onlinelibrary.wiley.com/doi/full-xml/10.1002/bmc.4728, type=“application/xml” (accessed on 28 October 2022)
r = opener.open(‘http://dx.doi.org/10.1111/jvp.13054’) print (r.info()[‘Link’])	https://onlinelibrary.wiley.com/doi/pdf/10.1111/jvp.13054, type=“application/pdf” (accessed on 28 October 2022) https://onlinelibrary.wiley.com/doi/full-xml/10.1111/jvp.13054, type=“application/xml” (accessed on 28 October 2022)
r = opener.open(‘http://dx.doi.org/10.1111/jphp.13353’) print (r.info()[‘Link’])	https://onlinelibrary.wiley.com/doi/pdf/10.1111/jphp.13353, type=“application/pdf” (accessed on 28 October 2022) https://onlinelibrary.wiley.com/doi/full-xml/10.1111/jphp.13353, type=“application/xml” (accessed on 28 October 2022)
r = opener.open(‘http://dx.doi.org/10.1111/jphp.12275’) print (r.info()[‘Link’])	http://academic.oup.com/jpp/article-pdf/66/10/1421/36221699/jphp12275.pdf, type=“application/pdf” (accessed on 28 October 2022)
r = opener.open(‘http://dx.doi.org/10.1211/jpp.59.8.0004’) print (r.info()[‘Link’])	http://academic.oup.com/jpp/article-pdf/58/4/449/36736506/jpp.58.4.0004.pdf, type=“application/pdf” (accessed on 28 October 2022)

**Table 6:** A comparative analysis of existing crawling systems or API Service Providers for the search queries, SQ (2): ‘Pharmacokinetics’, SQ (3) ‘Toxicology’, and SQ (4) ‘Neural network reduction’.

Web Crawler or API Service Provider	Number of Records: Metadata/Full-Text			Description and Notes
	PharmacoKinetics (SQ2)	Toxicology (SQ3)	Neural Network Reduction (SQ4)	
Scopus	1,228,515/38,4177	2,517,378/830,701	412,765/157,932	Facilitates source-unbiased metadata retrieval. However, 384,177 out of 1,228,515 metadata (SQ2), 830,701 out of 2,517,378 metadata (SQ3), and 157,932 out of 412,765 metadata (SQ4) can be retrieved as open-access full-text articles. https://www.scopus.com/search/form.uri?display=advanced. (accessed on 23 August 2022). Using article retrieval APIs, open-access full-text can be retrieved by DOI (document object identifier), PII (publication item identifier), EID (electronic identifier), Scopus ID, and Pubmed ID (Medline ID). https://dev.elsevier.com/documentation/FullTextRetrievalAPI.wadl. (accessed on 23 August 2022)
Springer	121,467	653,049	185,134	By using https://link.springer.com/advanced-search (accessed on 23 August 2022), 121467, 653049, and 185134 records are listed respectively for SQ2, SQ3, and SQ4. Automatic extraction of Springer Nature metadata and open-access full-text articles is possible using the Springer Nature API portal https://dev.springernature.com/ (accessed on 23 August 2022), provided we have a valid API key.
Crossref	70,573	315,269	1,698,727	Mostly supports itemized search with the title, author, DOI, ORCID ID, etc., for its metadata https://search.crossref.org/ (accessed on 26 August 2022), and Crossref REST API for metadata and full-text access in a more sophisticated way. https://www.crossref.org/documentation/retrieve-metadata/rest-api/text-and-data-mining-for-researchers/ (accessed on 26 August 2022).
Open Knowledge Maps	100 most relevant documents	100 most relevant documents	100 most relevant documents	A comprehensible visualization tool for bibliometric studies. However, in the given SQ2, SQ3, and SQ4, the outcome was limited to the 100 most relevant documents out of many. https://openknowledgemaps.org/ (accessed on 24 March 2023).
CiteseerX	87,696	150,117	4,908,661	A pioneer digital library that provides access to all open-access articles under one roof. Metadata extraction using web services was unsuccessful. However, full-text article downloads are labor-intensive. https://citeseerx.ist.psu.edu/ (accessed on 24 March 2023).
Web of Science	272,550/82,693	307,831/44,566	19,207/8747	Web of Science offers a large collection of citation databases while the coverage depends on the institution’s subscription depth. https://www.webofscience.com/wos/woscc/advanced-search (accessed on 18 October 2022). API calls also require a paid subscription for their metadata and full-text content downloads. https://developer.clarivate.com/apis (accessed on 18 October 2022).
Google Scholar	1,920,000	4,200,000	3,920,000	Relevant articles including open access and subscriptions were listed for the search keyword, while identifying and downloading full-text relevant articles for the search queries (SQ2, SQ3, and SQ4) appear to be labor-intensive.
PubMed	619,276/154,071	244,601/80,923	5135/2782	Search queries resulted in a total of 619110, 244491, and 5135 records with free full-text access of 154071, 80923, and 2782, respectively, for SQ2, SQ3, and SQ4. E-Summary, E-Fetch, and OAI-PMH services provide metadata content for PMCID or PMID. However, full-text downloads are labor-intensive. https://pubmed.ncbi.nlm.nih.gov/advanced/ (accessed on 24 March 2023).
PubMed Central	295,140	180,896	221,477	PubMed Central offered 295140, 180896, and 221,477 free full-text articles for SQ2, SQ3, and SQ4, respectively. https://www.ncbi.nlm.nih.gov/pmc/ (accessed on 24 March 2023). The search results are inclusive of MeSH terms while identifying relevant articles is labor-intensive. However, BioC API provides access to full-text content of all open-access articles, https://www.ncbi.nlm.nih.gov/research/bionlp/APIs/BioC-PMC/ (accessed on 24 March 2023), while other accessible PMC articles datasets include PMC Cloud Service, PMC OAI-PMH Service, PMC FTP Service, and E-Utilities. https://www.ncbi.nlm.nih.gov/pmc/tools/textmining/ (accessed on 24 March 2023).
IEEE Xplore	329	2302	14,700	With an institutional subscription, a total of 329, 2302, 14,700 scientific and technical articles published by IEEE were listed for SQ2, SQ3, and SQ4, respectively. https://ieeexplore.ieee.org/Xplore/home.jsp. (accessed on 24 March 2023) In addition, IEEE metadata API and dynamic query tool permit 200 API calls per day for an account with an institution ID. https://developer.ieee.org/io-docs (accessed on 24 March 2023).
PLOS	11,321	16,228	81,970	Listed a total of 11,321, 16,228, and 81,970 records for the SQ2, SQ3, and SQ4, respectively. https://journals.plos.org/plosone/search (accessed on 18 October 2022). In addition, Solr API provides access to the PLOS corpus of scientific articles. https://api.plos.org/solr/examples/ (accessed on 18 October 2022).
WCPK (Proposed Scheme)	1228,515/116,7089	2,517,378/2,391,509	412,765/392,126	When compared with other metadata and article services, the proposed WCPK facilitates automatic access to source-neutral metadata content through the Scopus metadata service. The full-text article retrieval gives a total of more than 95% through Scopus, Springer, and Crossref API services as well through journal home pages when these API calls are unsupportive.

## Data Availability

Our 1DATA and FARAD pages provide additional information and access to the source code of the project upon reasonable request. Please visit https://1data.life for any queries and contact information.

## Supplementary Materials

The following supporting information can be downloaded at: https://www.mdpi.com/article/10.3390/pharmaceutics15051384/s1, Table S1. Drugs classed code from ATC Classification and their corresponding drugs. Table S2. Metadata information collected for four different article types of the drug class QJ01–QJ05 from the SCOPUS search query is taken as a representative outcome. Table S3. Encoded results from TDM Crossref’s DOI Registration Agency Verification. Table S4. Encoded results from Crossref article full-text information.
