# Supplementary material for: An Automated Customizable Live Web Crawler for Curation of Comparative Pharmacokinetic Data: An Intelligent Compilation of Research-Based Comprehensive Article Repository
Source: Pharmaceutics. 2023 Apr 30;15(5):1384. doi: 10.3390/pharmaceutics15051384 (PMC10223110; doi:10.3390/pharmaceutics15051384)
Supplement: Supplementary file 1 [file pharmaceutics-15-01384-s001.zip › pharmaceutics-2272497-supplementary.pdf]

Supplementary Materials: An Automated Customizable Live Web Crawler for Curation of Comparative Pharmacokinetic Data: An Intelligent Compilation of Research-Based Comprehensive Article Repository

Remya Ampadi Ramachandran, Lisa A. Tell, Sidharth Rai, Nuwan Indika Millagaha Gedara, Xuan Xu, Jim E. Riviere and Majid Jaberi-Douraki

Table S1. Drugs classed code from ATC Classification and their corresponding drugs.

Drug Class	Drug names
J01A	Arestin, aureomycin, bristacycline, chlortetracycline, chlortetracycline and bisulfate, chlortetracycline and hydrochloride, clomocycline, declomycin, demeclocycline, demeclocycline and hydrochloride, demethylchlortetracycline, demethylchlortetracycline and hydrochloride, doxychel, doxycycline, doxycycline and calcium, doxycycline and fosfatex, doxycycline and hyclate, doxycycline and hydrate, doxycycline and hydrochloride, doxycycline and hydrochloride and hydrate, dynacin, eravacycline, eravacycline and dihydrochloride, lymecycline, lymepak, metacycline, methacycline, methacycline and hydrochloride, minocin, minocycline, minocycline and hydrochloride, monodox, nuzyra, omadacycline, omadacycline and tosylate, oracea, oxytetracycline, oxytetracycline and calcium, oxytetracycline and dihydrate, oxytetracycline and hydrochloride, penimepicycline, periostat, rolitetracycline, rolitetracycline and nitrate, rondomycin, sarecycline, sarecycline and hydrochloride, seysara, solodyn, sumycin, synterin, terramycin, tetracycline, tetracycline and hydrochloride, tetracycline and hydrochloride and epidihydrocholesterin, tetracycline and hydrochloride and hydrocortisone and acetate, tetracycline and metaphosphate, tetracycline and phosphate and complex, tetracycline and presteron, tetrax, tigecycline, tygacil, vibramycin, xerava
J01B	Amphicol, chloramphenicol, chloramphenicol and icn, chloramphenicol and palmitate, chloramphenicol and pantothenate and complex, chloramphenicol and sodium and succinate, chloramphenicol and stearate, chloramphenicol and succinate, chloromycetin, chloromycetin and palmitate, chloromycetin and succinate, econochlor, fluimucil and antibiotic, pantofenicol, quemicetina, thiamphenicol, thiamphenicol and aminoacetate and hydrochloride, thiamphenicol and glycinate and acetylcysteinate, thiamphenicol and glycinate and hydrochloride, thiocymetin, urfamycin
J01C	Amcill, amdinocillin, amdinocillin and pivoxil, amoxicillin, amoxicillin and clavulanate and potassium, amoxicillin andpotassium and clavulanate, amoxicillin and hydrate, amoxicillin and hydrate and potassium and clavulanate, amoxicillin andsodium, amoxicillin and trihydrate, amoxicilline, amoxil, ampicillin, ampicillin and cloxacillin, ampicillin andcloxacillin, ampicillin and dicloxacillin and sodium, ampicillin and dicloxacillin and sodium, ampicillin andbenzathine, ampicillin and hydrate, ampicillin and sodium, ampicillin and sodium and sulbactam and sodium, ampicillin and sodium andsulbactam and sodium, ampicillin and trihydrate, anhydrous and ampicillin, aseocillin, aspoxicillin, aspoxicillin and hydrate, augmentin, augpenin, azidocillin, azidocillin and sodium and salt, azlocillin, azlocillin and sodium, bacampicillin, bacampicillin and hydrochloride, bactocill, benzylpenicillin, benzylpenicillin and benzathine and hydrate, benzylpenicillin and benzathine and hydrate, benzylpenicillin and potassium, benzylpenicillin and sodium, bicillin and l-a, bicillin and l-a, calcipen, carbenicillin, carbenicillin anddisodium, carbenicillin and indanyl, carbenicillin and indanyl and sodium, carbenicillin and potassium, carbenicillin and sodium, carindacillin, carindacillin and sodium, clavulanic and acid and ticarcillin, clemizole and penicillin, clemizol-penicillin andgrunenthal, clometocillin, clometocillin and potassium, cloxacillin, cloxacillin and benzathine, cloxacillin and sodium, cloxacillin andsodium and hydrate, cloxapen, coactabs, coactin, dexacillin, dicloxacillin, dicloxacillin and sodium, dicloxacillin and sodium andhydrate, dispermox, duracillin, durapen, dycill, epicillin, floxacillin, floxapen, flucloxacillin, flucloxacillin and magnesium, flucloxacillin and sodium, flucloxacillin and sodium and hydrate, flucloxacillin and sodium and salt, geocillin, geopen, hetacillin, hetacillin and potassium, hetacin-k, infectobicillin and h-tabletten, lilacillin, longatren, mecillinam, melysin, metampicillin, metampicillin and sodium, methicillin and sodium, methicillin and sodium and monohydrate, meticillin and sodium, mezlin, mezlocillin, mezlocillin and sodium, mezlocillin and sodium and monohydrate, nafcillin, nafcillin and sodium, nafcillin and sodium and monohydrate, negaban, omnipen, omnipen-n, orbenin, oxacilina, oxacillin, oxacillin and sodium, oxacillin and sodium and hydrate, pasetocin, penamecillin, penglobe, penicillin and g, penicillin and g and benzathine, penicillin and g and benzathine, penicillin and g and potassium, penicillin and g and

	procaine, penicillin and g and sodium, penicillin and v, penicillin and v and benzathine, penicillin and v andbenzathine and tetrahydrate, penicillin and v and benzathine and tetrahydrate, penicillin and v and hydrabamine, penicillin and v andpotassium, pentcillin, peracin, permapen, permapen, pfizerpen, phenethicillin, phenethicillin and potassium, pheneticillin, pheneticillin and potassium, phenoxymethylpenicillin, phenoxymethylpenicillin and benzathine, phenoxymethylpenicillin and benzathine, phenoxymethylpenicillin and calcium, phenoxymethylpenicillin and potassium, piperacillin, piperacillin and hydrate, piperacillin andsodium, piperacillin and sodium and tazobactam, pipracil, pivampicillin, pivampicillin and hydrochloride, pivampicillin and pamoate, pivampicillin and probenate, pivmecillinam, pivmecillinam and hydrochloride, pondocillin, propicillin, propicillin and potassium, rixapen, serfabiotic, spectrobid, staphcillin, sulbactam, sulbactam and benzathine, sulbactam and pivoxil, sulbactam and sodium, sulbenicillin, sulbenicillin and sodium, sultamicillin, sultamicillin and tosilate and hydrate, sultamicillin and tosylate and dihydrate, syncillin, synthepen, talampicillin, talampicillin and hydrochloride, tazobactam, tazobactam and piperacillin, tazobactam and sodium, tazocin, tegopen, temocillin, temocillin and disodium and salt, ticarcillin, ticarcillin and cresyl and sodium, ticarcillin and disodium, ticarcillin and monosodium, ticarcillin and monosodium and hydrate, ticarcillin and sodium, ticillin, timentin, unasyn, unasyn-s, unasyn-s, unipen, veetids, versapen, viccillin, viccillin and s, viccillin and s, wycillin, wycillin
J01D	Alenfral, amasulin, anspor, avycaz, azactam, aztreonam, aztreonam and lysine, bestcall, biapenem, carbenin, carumonam, carumonam andsodium, cayston, ceclor, cefacetrile, cefacetrile and sodium, cefaclor, cefaclor and anhydrous, cefaclor and monohydrate, cefadroxil, cefadroxil and monohydrate, cefadyl, cefalexin, cefaloridine, cefalotin, cefalotin and sodium, cefalotina and fabra, cefamandole, cefamandole and nafate, cefamandole and sodium, cefapirin, cefapirin and benzathine, cefapirin and sodium, cefatrizine, cefazedone, cefazolin, cefazolin and sodium, cefazolin and sodium and hydrate, cefbuperazone, cefbuperazone and sodium, cefcapene, cefcapene and pivoxil and hydrochloride and hydrate, cefdinir, cefdinir and monohydrate, cefditoren, cefditoren and pivoxil, cefepime, cefepime and dihydrochloride and hydrate, cefepime and hydrochloride, cefetamet, cefetamet and pivoxil and hydrochloride, cefiderocol, cefiderocol and sulfate and tosilate, cefiderocol and sulfate and tosylate, cefiderocol and tosilate and sulfate and hydrate, cefixime, cefixime and hydrate, cefixime and trihydrate, cefizox, cefmenoxime, cefmenoxime and hydrochloride, cefmetazole, cefmetazole and sodium, cefminox, cefminox and sodium and hydrate, cefobid, cefodizime, cefodizime and sodium, cefonicid, cefonicid and monosodium, cefonicid and sodium, cefoperazone, cefoperazone and sodium, cefoperazone and sodium and sulbactam and sodium, ceforanide, cefotan, cefotaxim and hikma, cefotaxime, cefotaxime and sodium, cefotetan, cefotetan and disodium, cefotetan and sodium, cefotiam, cefotiam and hexetil and hydrochloride, cefotiam and hydrochloride, cefoxitin, cefoxitin and sodium, cefozopran, cefozopran and hydrochloride, cefpiramide, cefpiramide and sodium, cefpirome, cefpirome and sulfate, cefpodoxime, cefpodoxime and proxetil, cefprozil, cefprozil and monohydrate, cefradine, cefrom, cefroxadine, cefroxadine and hydrate, cefsulodin, cefsulodin and sodium, ceftaroline and fosamil, ceftazidime, ceftazidime and avibactam, ceftazidime and hydrate, ceftazidime and pentahydrate, cefteram, cefteram and pivoxil, ceftezole, ceftezole and sodium, cefthan-ds, ceftibuten, ceftibuten and dihydrate, ceftibuten and hydrate, ceftin, ceftizoxime, ceftizoxime and alapivoxil and hydrochloride, ceftizoxime and sodium, ceftobiprole and medocaril, ceftolozane and sulfate and tazobactam and sodium, ceftriaxone, ceftriaxone and sodium, ceftriaxone and sodium and hydrate, cefuroxime, cefuroxime and axetil, cefuroxime and pivoxetil, cefuroxime and sodium, cefzil, cefzon, celoslin, celtol, cephacetrile, cephacetrile and sodium, cephalexin, cephalexin and hydrochloride, cephalexin and monohydrate, cephaloridine, cephalothin, cephalothin and sodium, cephapirin, cephapirin and benzathine, cephapirin and sodium, cephradine, ceptaz, ceradon, claforan, doribax, doripenem, doripenem and hydrate, doripenem and monohydrate, duricef, epocelin, epoxim, ertapenem, ertapenem and sodium, falomesin, faropenem and medoxomil, faropenem and sodium and hydrate, fetcroja, fetroja, firstcin, flomox, flomoxef, flomoxef and sodium, flumarin, fortaz, imipenem and cilastatin and sodium, imipenem, and cilastatin and relebactam, invanz, kefdole, keflex, keflin, kefloridin, keftab, keiperazon, kenicef, latamoxef, latamoxef and sodium, lorabid, loracarbef, loracarbef and monohydrate, lorbef, mandol, maxipime, mefoxin, meiact, meicelin, meronem, meropenem, meropenem and hydrate, meropenem and vaborbactam, merrem, merrem and i.v., metricure, monocef, monocid, moxalactam and disodium, neucef, omegacin, omnicef, orapenem, oraspor, oroken, panipenem and betamipron, panixine and disperdose, pansporin, pansporin-t, peracef, precef, primaxin, propylene and glycolate and cefatrizine, recarbrio, rocephin, seapuron, seftem, shiomarin, spectracef, sulperazon, sumacef, suncefal, suprax, takesulin, tazicef, tebipenem and pivoxil, tebipenem and pivoxil and hydrobromide, teflaro, tomiron, vabomere, vantin, velosef, vetrimast, zavicefta, zefazone, zerbaxa, zinacef
J01E	Trimethoprim, proloprim, trimpex, trimethoprim and sulfate, trimethoprim and lactate, wellcoprim, trimethoprim and hydrochloride, primsol, brodimoprim, iclaprim, iclaprim and mesylate, sulfisomidine,

	sulfaisodimidine, sulfamethizole, thiosulfil, sulfamethazine, sulfadimidine, sulfamezathine, sulfadimidine and sodium, sulfamethazine and sodium and salt, intradine, sulfadimidin, sulfapyridine, sulfisoxazole, sulfafurazole, gantrisin, sulfisoxazole and acetyl, acetylsulfisoxazole, sulfisoxazole and diolamine, sulfafurazole and diolamine, sulfanilamide, sulfamine, streptocid, sulfathiazole, sulfathiourea, sulphathiourea, sulfamethoxazole, gantanol, acetylsulfamethoxazole, sulfadiazine, and silver, sulfadiazine and silver, silvadene, sulfadiazine, sulfapyrimidine, sulfadiazine and sodium, sulfamoxole, sulfadimethoxine, agribon, sulfadimethoxine and sodium, bactotril, sulfalene, sulfamethopyrazine, kelfizina, sulfametomidine, sulfametomidine and monohydrate, sulfameter, sulfametoxydiazine, sulfamethoxypyrimidine, sulfamethoxypyridazine, lederkyn, sulfamethoxypyridazine and sodium and salt, avemix, sulfaperin, sulfamerazine, sulfaphenazole, sulphaphenazole, sulfabid, sulfamazone, sulfamethoxazole and trimethoprim, bactrim, septra
J01F	Acetylspiramycin, azasite, azimycin, azithromycin, azithromycin and dihydrate, azithromycin and hydrate, berythromycin, biaxin, bristamycin, clarithromycin, clarithromycin and lactobionate, cleocin, cleocin and hydrochloride, cleocin and pediatric, clindamycin, clindamycin and hydrochloride, clindamycin and palmitate and hydrochloride, clindamycin and phosphate, clindamycin and phosphate andhydrate, cofamix and sts, dalacin, dalacin-s, dirithromycin, dynabac, erasis, erygel, eryped, erysec, erythrocin, erythrocin and w, erythromycin, erythromycin and acistrate, erythromycin and b, erythromycin and estolate, erythromycin and estorate, erythromycin andethylsuccinate, erythromycin and gluceptate, erythromycin and glucoheptonate, erythromycin and lactobionate, erythromycin andpropionate, erythromycin and salnacedin, erythromycin and salnacedin and dihydrate, erythromycin and stearate, erythromycin and stinoprate, erythromycin and thiocyanate, evoclin, flurithromycin, ilosone, ilotycin and gluceptate, josamy, josamycin, josamycin and propionate, klacid, leucomycin and a3, lincocin, lincomycin, lincomycin and hydrochloride and hydrate, matromycin, medemycin, midecamycin, midecamycinand acetate, miocamycin, oleandomycin and phosphate, pristinamycin, quinupristin and dalfopristin, ricamycin, rokitamycin, rovamycin, rovamycina, roxithromycin, rulide, solithromycin, spiramaicina, spiramycin, spiramycin and adipate, spiramycin and embonate, spiramycin and ii, stafac, staticin, synercid, telithromycin, triacetyloleandomycin, troleandomycin, virginiamycin, zithromac
J01G	Amikacin, amikacin and sulfate, amikin, arbekacin, arbekacin and sulfate, bekanamycin, bekanamycin and sulfate, bethkis, dibekacin, dibekacin and sulfate, fradiomycin, fradiomycin and sulfate, garamycin, gentacin, gentamicin, gentamicin and sulfate, habekacin, isepacin, isepamicin, isepamicin and sulfate, kanamycin and b, kanamycin and monosulfate, kanamycin and sulfate, kanendomycin, kantrex, kaomycine, mycifradin, nebcin, neomycin, neomycin and palmitate, neomycin and sulfate, neomycin and undecylenate, netilmicin, netilmicin and sulfate, netira, netromycin, panimycin, plazomicin, plazomicin and sulfate, ribostamycin, ribostamycin and sulfate, siseptin, sisomicin, sisomicin and sulfate, sofratulle, streptoduocin, streptomycin, streptomycin and sulfate, streptomycin and sulfate and dihydrostreptomycin and sulfate, tobracin, tobramycin, tobramycin and sulfate, tobrex, vistamycin, zemdri
J01M	Apurone, aqualinic, avelox, avelox and iv, baxdela, ciloxan, cinobac, cinoxacin, ciprofloxacin, ciprofloxacin and hemiheptahydrate, ciprofloxacin and hydrate, ciprofloxacin and hydrochloride, ciprofloxacin and hydrochloride and hydrate, ciprofloxacin and lactate, ciproxin, cravit, delafloxacin, delafloxacin and meglumine, enoxacin, enoxacin and hydrate, factiv, factive, fleroxacin, floxin, flumequine, garenoxacin and mesilate and hydrate, garenoxacin and mesylate, gatiflo, gatifloxacin, gatifloxacin and anhydrous, gatifloxacinand hydrate, gemifloxacin, gemifloxacin and mesylate, grepafloxacin and hydrochloride, karunomazin, lascufloxacin and hydrochloride, lasvic, levaquin, levofloxacin, levofloxacin and hemihydrate, levofloxacin and hydrate, levonadifloxacin, lomefloxacin, lomefloxacin and hydrochloride, lomefloxacin and mesylate, maxaquin, megalocin, megalone, moxifloxacin, moxifloxacin and hydrochloride, nalidixate and sodium, nalidixate and sodium and monohydrate, nalidixic and acid, neggram, norfloxacin, norfloxacin and nicotinate, noroxin, ofloxacin, ofloxacin and hydrochloride, omniflox, otiprio, oxolinic and acid, panacid, pazufloxacin, pazufloxacin and mesilate, pazufloxacin and methanesulfonate, pefloxacin, pefloxacin and mesylate, penetrex, pipemidic and acid, pipemidic and acid and hydrate, piromidic and acid, proquin and xr, prulifloxacin, quinabic and soluble and powder, quixin, rosoxacin, roxadyl, rufloxacin, rufloxacin and hydrochloride, sitafloxacin, sitafloxacin and hydrate, sparfloxacin, tarivid, temafloxacin and hydrochloride, tequin, tosufloxacin, tosufloxacin and tosilate, tosufloxacin and tosylate and hydrate, tosuxacin, trovafloxacin, trovafloxacin and mesylate, trovan, vigamox, zymaxid
J01R	Amoxicillin and clavulanate and potassium, amoxicillin and hydrate and potassium and clavulanate, amoxicillin and hydrate, and clarithromycin and lansoprazole, amoxicillin and potassium and clavulanate, ampicillin and cloxacillin, ampicillin and dicloxacillin and sodium, ampicillin and sodium and sulbactam and sodium, augmentin, augpenin, clavulanic and acid and ticarcillin, lampion, lansap, lansoprazole, and

	amoxicillin and hydrate and metronidazole, piperacillin and sodium and tazobactam, prevpac, tazobactam and piperacillin, tazocin, timentin, unasyn-s, viccillin and s
J01X	Adspec, aerosporin, baciim, bacitracin, bacitracin and methylenedisalicylate, bacitracin and zinc, clofoctol, colistimethate and sodium, colistin, colistin and sodium and methanesulfonate, colistin and sulfate, colymycin and s, cubicin, dalbavancin, dalvance, daptomycin, diethanolamine and fusidate, elyzol, flagyl, flagyl and i.v., fosfocin, fosfomycin, fosfomycin and calcium, fosfomycin and calcium and hydrate, fosfomycin and sodium, fosfomycin and tromethamine, fosmicin, fosmicin and s, fucidin, fucidin and leo, fucithalmic, furadantin, furagin, furazidin, fusidate and sodium, fusidic and acid, fusidic and acid and diolamine, fusidic and acid and hemihydrate, haisigyn, hexamine, hexamine and mandelate, hiprex, lefamulin, lefamulin and acetate, linezolid, macrobid, mandelamine, methacolimycin, methenamine, methenamine and hippurate, methenamine and mandelate, methenamine and sulfosalicylate, metronidazole, metronidazole and benzoate, metronidazole and hydrochloride, metronidazole and phosphate, monurol, nifurtoinol, nitrofurantoin, nitroxoline, noritate, nuvessa, octofene, orbactiv, oritavancin and diphosphate, ornidazole, phosphonomycin, polymixin and b and sulfate, polymyxin and b, polymyxin and b and sulfate, polysporin, prospec, sivextro, sodium and fusidate, sogecoli, spectinomycin, spectinomycin and hydrochloride, spectinomycin and hydrochloride and hydrate, tagocid, tedizolid, tedizolid and phosphate, teicoplanin, telavancin and hydrochloride, tindamax, tinidazole, torezolid, torezolid and phosphate, trobicin, urfadyn, uropurat, vancocin, vancoled, vancomycin, vancomycin and hydrochloride, vandazole, vibativ, xenleta, xibornol
J02A	Ambisome, amphotec, amphotericin and b, ancobon, anidulafungin, cancidas, caspofungin, caspofungin and acetate, cresemba, diflucan, eraxis, fluconazole, flucytosine, fosfluconazole, fungizone, funguard, hachimycin, isavuconazole, itraconazole, itrizole, ketoconazole, micafungin, micafungin and sodium, micafungin and sodium and hydrate, micatin, miconazole, miconazole and nitrate, monistat, mycamine, nizoral, noxafil, oravig, posaconazole, prodif, sporanox, trichomycin, voriconazole, xolegel
J04A	Aemcolo, aminosalicylate and sodium, aminosalicylate and sodium, aminosalicylic and acid, amithiozone, bedaquiline, bedaquiline and fumarate, calcium and para-aminosalicylate, capastat and sulfate, capreomycin, capreomycin and sulfate, citazone, cycloserine, delamanid, deltyba, enviomycin, enviomycin and sulfate, ethambutol, ethambutol and hydrochloride, ethionamide, fenamisal, isoniazid, isoniazid and calcium and pyruvinate, isoniazid and glucuronate and sodium, isoniazid and sodium and methanesulfonate, isoniazid and sodium and methanesulfonate and hydrate, isoniazid and sodium and methanesulfonate and monohydrate, laniazid, morinamide, myambutol, mycobutin, neoiscotin, nippas and calcium, pamisyl, parasal and sodium, parasal and sodium, phenyl and aminosalicylate, pretomanid, priftin, prothionamide, protionamide, pyrazinamide, pyruvic and acid and calcium and isoniazid, rifabutin, rifadin, rifadine, rifampicin, rifampicin and sodium, rifampin, rifampin and isoniazid, rifampin, and isoniazid and pyrazinamide, rifamycin, rifamycin and sodium, rifamycin and sv, rifamycin and sv and sodium and salt, rifapentine, rifater, rifocina, rimactane, seromycin, servambutol, sirturo, terivalidin, terizidone, thioacetazone, tiocarlide, trecator, trevintix, tuberactin
J04B	Aczone, aldesulfone and sodium, clofazimine, dapsone, diaphenylsulfone, diasone and sodium, lamprene, sulfoxone and sodium
J05A	Abacavir, abacavir and succinate, abacavir and sulfate, abacavir and sulfate and lamivudine, abacavir, and lamivudine and zidovudine, aciclovir, acyclovir, acyclovir and sodium, adefovir and dipivoxil, adefovir and pivoxil, agenerase, agucort, amenalief, amenamevir, amprenavir, aptivus, asunaprevir, atazanavir, atazanavir and cobicistat, atazanavir and sulfate, atripla, avigan, baloxavir and marboxil, baraclude, bictegravir, and emtricitabine and tenofovir and alafenamide, biktarvy, boceprevir, brivudine, bulevirtide, bulevirtide andacetate, cabotegravir, cabotegravir and sodium, camvia, cidofovir, cidofovir and dihydrate, cimduo, clevudine, combivir, complera, copegus, crixivan, cytovene, cytovene and iv, daclatasvir, daclatasvir and dihydrochloride, daclatasvir and hydrochloride, daclatasvir, andasunaprevir and beclabuvir, daklinza, darunavir, darunavir and cobicistat, darunavir and ethanolate, darunavir, and cobicistat, andemtricitabine and tenofovir and alafenamide, dasabuvir, dasabuvir and sodium and hydrate, dasabuvir and sodium and monohydrate, dasabuvir, and ombitasvir, and paritaprevir and ritonavir, delavirdine, delavirdine and mesilate, delavirdine and mesylate, delstrigo, denavir, dendrid, descovy, didanosine, dolutegravir, dolutegravir and lamivudine, dolutegravir and sodium, dolutegravir and sodium andrilpivirine and hydrochloride, dolutegravir, and abacavir and lamivudine, doravirine, doravirine, and lamivudine and tenofovir anddisoproxil, dovato, dutrebis, ebilfumin, edurant, efavirenz, efavirenz, and emtricitabine and tenofovir and disoproxil, efavirenz, andlamivudine and tenofovir and disoproxil and fumarate, elbasvir, elbasvir and grazoprevir, elvitegravir, elvitegravir, and cobicistat,andemtricitabine and tenofovir and alafenamide, elvitegravir, and cobicistat, and emtricitabine and tenofovir and disoproxil, emtricitabine, emtricitabine and tenofovir and alafenamide, emtricitabine and tenofovir and disoproxil, emtricitabine, and rilpivirine andtenofovir and alafenamide, emtriva, enfuvirtide, enisamium and iodide, entecavir, entecavir and hydrate,

	epclusa, epivir, epzicom, erelsa, etravirine, eviplera, evotaz, exviera, faldaprevir and sodium, famciclovir, famvir, favipiravir, flumadine, fortovase, fosamprenavir, fosamprenavir and calcium, fosamprenavir and calcium and hydrate, fosamprenavir and sodium, foscarnet and sodium, foscarnetand sodium and hydrate, foscavir, fosfonet and sodium, fostemsavir, fostemsavir and trometamol, fostemsavir and tromethamine, fuzeon, ganciclovir, ganciclovir and sodium, genvoya, glecaprevir and pibrentasvir, grazoprevir, grazoprevir and anhydrous, grazoprevir andhydrate, grazyna, harvoni, hepcludex, hepsera, herplex, ibalizumab, ibalizumab-uiyk, idoxuridine, incivek, incivo, indinavir, indinavirand hydrate, indinavir and sulfate, indinavir and sulfate and ethanolate, inosine and pranobex, intelence, invirase, isentress, isoprinosine, juluca, kaletra, lamivudine, lamivudine and raltegravir, lamivudine and raltegravir and potassium, lamivudine and tenofovirand disoproxil, lamivudine and zidovudine, lamivudine, and nevirapine and zidovudine, laninamivir, laninamivir and octanoate andhydrate, ledipasvir and sofosbuvir, letermovir, lexiva, lopinavir and ritonavir, lysozyme, lysozyme and hydrochloride, maraviroc, marboran, maribavir, mavyret, methisazone, metisazone, moroxydine, mucozome, muramidase, nelfinavir, nelfinavir and mesilate, nelfinavirandmesylate, nevirapine, norvir, odefsey, olysio, ombitasvir, and paritaprevir and ritonavir, oseltamivir, oseltamivir andphosphate, penciclovir, penciclovir and sodium, pentanedioic and acid and imidazolyl and ethanamide, peramivir, peramivir and hydrate, pifeltro, pleconaril, prevymis, prezcobix, prezista, raltegravir, raltegravir and potassium, rapivab, rebetol, rekambys, relenza, rescriptor, retrovir, reyataz, rezolsta, ribasphere, ribavirin, rilpivirine, rilpivirine and hydrochloride, rilpivirine and hydrochloride,and tenofovir and disoproxil and fumarate and emtricitabine, rimantadine, rimantadine and hydrochloride, ritonavir, rukobia, sanilvudine, saquinavir, saquinavir and mesilate, saquinavir and mesylate, selzentry, simeprevir, simeprevir and sodium, sitavig, sofosbuvir, sofosbuvir and velpatasvir, sofosbuvir, and velpatasvir and voxilaprevir, sovaldi, sovriad, stavudine, stribild, sunvepra, sustiva, symtuza, tamiflu, technivie, tecovirimat, tecovirimat and monohydrate, telaprevir, telbivudine, telzir, temixys, tenofovir andalafenamide, tenofovir and alafenamide and fumarate, tenofovir and disoproxil and fumarate, tenofovir and disoproxil and maleate, tenofovir and disoproxil and phosphate, tilorone and hydrochloride, tipranavir, tipranavir and disodium, tivicay, trisodium andphosphonoformate and hexahydrate, triumeq, trizivir, trogarzo, tromantadine, tromantadine and hydrochloride, truvada, tyzeka, umifenovir, valaciclovir, valaciclovir and hydrochloride, valaciclovir and hydrochloride and hydrate, valacyclovir, valacyclovir andhydrochloride, valcyte, valganciclovir, valganciclovir and hydrochloride, valtrex, vemlidy, victrelis, vidarabine, vidarabine andanhydrous, vidarabine and monohydrate, vidarabine and phosphate, vidarabine and sodium and phosphate, viekira and pak, viracept, viramune, virazole, viread, vistide, vitekta, vitrasert, vocabria, vosevi, ximency, xofluza, zalcitabine, zanamivir, zanamivir and hydrate, zepatier, ziagen, zidovudine, zidovudine and lamivudine, zirgan, zostex, zovirax

Table S2. Metadata information collected for four different article types of the drug class QJ01 - QJ05 from the SCOPUS search query is taken as a representative outcome.

Extracted data from Scopus metadata	Examples – Original Research Article, Review Article, Book Chapter, Book			
eid	2-s2.0-85131400463	2-s2.0-85131079917	2-s2.0-85127125813	2-s2.0-85131987438
doi	10.1016/j.ejps.2022.106219	10.3390/antibiotics11050701	10.1016/B978-0-12-818902-3.00015-4	10.1002/9781119497813
pii	S092809872200104X			
pubmed_id	35618200			
title	Amikacin pharmacokinetics in elderly patients with severe infections	Ways to Improve Insights into Clindamycin Pharmacology and Pharmacokinetics Tailored to Practice	Impact of pregnancy on maternal pharmacokinetics of medications	PHYSIOLOGICALLY BASED PHARMACOKINETIC (PBPK) MODELING AND SIMULATIONS: Principles, Methods, and Applications in the Pharmaceutical Industry, Second Edition
subtype	ar	re	ch	bk
subtypeDescription	Article	Review	Book Chapter	Book
creator	Medellín-Garibay S.E.	Álvarez L.A.	Ryu R.	Peters S.A.
afid	60032541;60031335;60025844;60016574	60121207;60068577;60032114;60031069;60025063	60015481	60018366
affilname	Hospital Severo Ochoa;Universidad Autonoma de San Luis Potosi;Hospital Universitario Puerta de Hierro Majadahonda;Universidad Complutense de Madrid, Facultad de Farmacia	Departement Microbiologie, Immunologie en Transplantatie;Rega Institute for Medical Research;Erasmus MC;KU Leuven– University Hospital Leuven;KU Leuven	University of Washington	Merck KGaA
affiliation_city	Leganes;San Luis Potosí;Majadahonda;Madrid	Leuven;Leuven;Rotterdam;3000 Leuven;3000 Leuven	Seattle	Darmstadt
affiliation_country	Spain;Mexico;Spain;Spain	Belgium;Belgium;Netherlands;Belgium;Belgium	United States	Germany
author_count	8	7	2	1
author_names	Medellín-Garibay, Susanna E.;Romano-Aguilar, Melissa;Parada, Alejandro;Suárez, David;Romano-Moreno, Silvia;Barcia, Emilia;Cervero, Miguel;García, Benito	Álvarez, Laura Armengol;Van de Sijpe, Greet;Desmet, Stefanie;Metsemakers, Willem Jan;Spriet, Isabel;Allegaert, Karel;Rozenski, Jef	Ryu, Rachel;Hebert, Mary F.	Peters, Sheila Annie

author_ids	36610623800;57210937049;57729583200;57730179100;57218390495;6603720469;57222997862;49761285500	57720611100;57194831175;35740187100;23981026600;15127963300;7003304865;7006175622	56030568100;7102121868	37019415800
author_afids	60031335;60031335;60031335;60032541-60025844;60031335;60016574;60032541-60025844;60032541-60025844	60068577;60025063-60031069;60031069-60121207;60025063-60031069;60025063-60031069;60025063-60032114;60068577	60015481;60015481	60018366
coverDate	8/1/2022	5/1/2022	1/1/2021	1/1/2021
coverDisplayDate	8/1/2022	May-2 2022	1/1/2021	1/1/2021
publicationName	European Journal of Pharmaceutical Sciences	Antibiotics	Clinical Pharmacology During Pregnancy	Physiologically-Based Pharmacokinetic (PBPK) Modeling and Simulations: Principles, Methods, and Applications in the Pharmaceutical Industry, Second Edition
issn	9280987			
source_id	21331	21100469670	21101082434	21101092507
eIssn	18790720	20796382		
aggregationType	Journal	Journal	Book	Book
volume	175	11		
issueIdentifier		5		
article_number	106219	701		
pageRange			19-46	1-591
description	Objective: The aim of this study was to characterize the population pharmacokinetics of amikacin in elderly patients by means of nonlinear mixed effects modelling and to propose initial dosing schemes to optimize therapy based on PK/PD targets. Method: A total of 137 elderly patients from 65 to 94 years receiving intravenous amikacin and routine therapeutic drug monitoring at Hospital Universitario Severo Ochoa were included. Concentration–time data and clinical information were retrospectively collected;	Given the increase in bacterial resistance and the decrease in the development of new antibiotics, the appropriate use of old antimicrobials has become even more compulsory. Clindamycin is a lincosamide antibiotic approved for adults and children as a drug of choice for systemic treatment of staphylococcal, streptococcal, and gram-positive anaerobic bacterial infections. Because of its profile and high bioavailability, it is	The efficacy and safety of medications during pregnancy is highly variable and often different than in the nonpregnant population. In part, this variability can be explained by changes in pharmacokinetics. This chapter discusses many of the physiologic changes that occur	Physiologically Based Pharmacokinetic (PBPK) Modeling and Simulations The first book dedicated to the emerging field of physiologically based pharmacokinetic modeling (PBPK) Now in its second edition, Physiologically Based Pharmacokinetic (PBPK) Modelling and Simulations: Principles, Methods, and Applications in the Pharma Industry remains the premier reference book throughout the rapidly growing PBPK user community. Using clear and concise

	initial doses of amikacin ranged from 5.7 to 22.5 mg/kg/day and each patient provided between 1 and 10 samples. Results: Amikacin pharmacokinetics were best described by a two-compartment open model; creatinine clearance (CrCL) was related to drug clearance (2.75 L/h/80 mL/min) and it was augmented 28% when non-steroidal anti-inflammatory drugs were concomitantly administered. Body mass index (BMI) influenced the central volume of distribution (17.4 L/25 kg/m2). Relative absolute prediction error was reduced from 33.2% (base model) to 17.9% (final model) when predictive performance was evaluated with a different group of elderly patients. A nomogram for initial amikacin dosage was developed and evaluated based on stochastic simulations considering final model to achieve PK/PD targets (Cmax/MIC>10 and AUC/MIC>75) and to avoid toxic threshold (Cmin<2.5 mg/L). Conclusion: Initial dosing approach for amikacin was designed for elderly patients based on nonlinear mixed effects modeling to maximize the probability to attain efficacy and safety targets considering individual BMI and CrCL.	commonly used as part of an oral multimodal alternative for prolonged parenteral antibiotic regimens, e.g., to treat bone and joint or prosthesis-related infections. Clindamycin is also frequently used for (surgical) prophylaxis in the event of beta-lactam allergy. Special populations (pediatrics, pregnant women) have altered cytochrome P450 (CYP)3A4 activity. As clindamycin is metabolized by the CYP3A4/5 enzymes to bioactive N-demethyl and sulfoxide metabolites, knowledge of the potential relevance of the drug's metabolites and disposition in special populations is of interest. Furthermore, drug–drug interactions derived from CYP3A4 inducers and inhibitors, and the data on the impact of the disease state on the CYP system, are still limited. This narrative review provides a detailed survey of the currently available literature on pharmacology and pharmacokinetics and identifies knowledge gaps (special patient population, drug–drug, and drug–disease interactions) to describe a research strategy for precision medicine.	during pregnancy and their impact on medication pharmacokinetics. Changes in volume of distribution, protein binding, blood flow, renal filtration, drug metabolizing enzymes (e.g., CYP3A, CYP2D6, CYP2C9, uridine diphosphate glucuronyltransferase, CYP1A2, and CYP2C19) and transporters (e.g., p-glycoprotein) are discussed. Taking into account and adjusting for the pharmacokinetic changes that occur during pregnancy will help to minimize the variability in patient response. This approach is particularly important for medications with narrow therapeutic ranges. Although critically important, pharmacokinetic changes should be taken as only one component in determining optimum medication selection and dosage.	language, author Sheila Annie Peters connects theory with practice as she explores the vast potential of PBPK modeling for improving drug discovery and development. This fully updated new edition covers key developments in the field of PBPK modelling and simulations that have emerged in recent years. A brand-new section provides case studies in different application areas of PBPK modelling, including drug-drug interaction, genetic polymorphism, renal impairment, and pediatric extrapolation. Additional chapters address topics such as model-informed drug development (MIDD) and expose readers to a wide range of current applications in the field. Throughout the book, substantially revised chapters simplify complex topics and offer a balanced view of both the opportunities and challenges of PBPK modelling. Providing timely and comprehensive coverage of one of the most exciting new areas of pharmaceutical science, this book: Describes the principles behind physiological modeling of pharmacokinetic processes, inter-individual variability, and drug interactions for small molecule drugs and biologics Features a wealth of new figures and case studies of the applications of PBPK modelling along the value chain in drug discovery and development Reflects the latest regulatory guidelines on the reporting of PBPK modelling analysis Includes

				access to a new companion website containing code, datasets, explanations of case examples in the text, and discussion of key developments in the field Contains a brief overview of the field, end-of-chapter keywords for easy reference, and an extensive bibliography Physiologically Based Pharmacokinetic (PBPK) Modeling and Simulations: Principles, Methods, and Applications in the Pharmaceutical Industry, Second Edition is an indispensable -single-volume resource for beginning and intermediate practitioners across the pharmaceutical sciences in both industry and academia.
authkeywords	Antiinfectives | Clinical pharmacokinetics | Individualized drug therapy | Pharmacometrics | Population pharmacokinetics | Special populations | Therapeutic drug monitoring	antibiotic | bacterial infections | clindamycin | CYP450 enzymes | drug–drug interactions | pharmacokinetics | special patient populations	AUC | Bioavailability | Blood flow | Clearance | Enzymes | Half-life | Metabolism | Pharmacokinetics | Pregnancy | Protein binding | Transporters | Volume	
citedby_count	0	1	0	1
openaccess	1	1	0	0
freetoread	publisherfullgold	repositoryvor		
freetoreadLabel	Gold	Green		
fund_acr				
fund_no	undefined	undefined	undefined	undefined
fund_sponsor				

Table S3. Encoded results from TDM Crossref's DOI Registration Agency Verification.

**DOI Registration Agency Verification:**
https://api.crossref.org/works/10.12998/wjcc.v10.i18.6218/agencyb'{"status":"ok","message-type":"work-agency","messageversion":"1.0.0","message":{"DOI":"10.12998\\/wjcc.v10.i18.6218","agency":{"id":"crossref","label":"Crossref"}}}'<http://dx.doi.org/10.12998/wjcc.v10.i18.6218>; rel="canonical", <https://www.wjgnet.com/2307-8960/full/v10/i18/6218.htm>; version="vor"; rel="item"
https://api.crossref.org/works/10.22038/ijp.2017.26942.2320/agencyb'{"status":"ok","message-type":"work-agency","messageversion":"1.0.0","message":{"DOI":"10.22038\\/ijp.2017.26942.2320","agency":{"id":"medra","label":"mEDRA"}}}'
https://api.crossref.org/works/10.3760/cma.j.issn.2095-4352.2019.11.001/agencyb'{"status":"ok","message-type":"work-agency","message-version":"1.0.0","message":{"DOI":"10.3760\\/cma.j.issn.2095-4352.2019.11.001","agency":{"id":"istic","label":"ISTIC"}}}'

Table S4. Encoded results from Crossref article full-text information.

**Crossref article full-text information:**
<http://dx.doi.org/10.1111/jvp.13054>; rel="canonical", <https://onlinelibrary.wiley.com/doi/pdf/10.1111/jvp.13054>; version="vor"; type="application/pdf"; rel="item", <https://onlinelibrary.wiley.com/doi/full-xml/10.1111/jvp.13054>; version="vor"; type="application/xml"; rel="item", <https://onlinelibrary.wiley.com/doi/pdf/10.1111/jvp.13054>; version="vor"; rel="item", <http://onlinelibrary.wiley.com/termsAndConditions#vor>; version="vor"; rel="license", <http://doi.wiley.com/10.1002/tdm_license_1.1>; version="tdm"; rel="license", <http://orcid.org/0000-0003-3657-4703>; title="Mario Giorgi"; rel="author", <http://orcid.org/0000-0001-5322-3281>; title="Amnart Poapolathep"; rel="author", <http://orcid.org/0000-0002-5249-903X>; title="Saranya Poapolathep"; rel="author"
<http://dx.doi.org/10.1111/jphp.13353>; rel="canonical", <https://api.wiley.com/onlinelibrary/tdm/v1/articles/10.1111%2Fjphp.13353>; version="vor"; type="application/pdf"; rel="item", <https://onlinelibrary.wiley.com/doi/pdf/10.1111/jphp.13353>; version="vor"; type="application/pdf"; rel="item", <https://onlinelibrary.wiley.com/doi/full-xml/10.1111/jphp.13353>; version="vor"; type="application/xml"; rel="item", <http://academic.oup.com/jpp/article-pdf/72/12/1750/36589809/jphp13353.pdf>; version="vor"; type="application/pdf"; rel="item", <http://academic.oup.com/jpp/article-pdf/72/12/1750/36589809/jphp13353.pdf>; version="vor"; rel="item", <https://academic.oup.com/journals/pages/open_access/funder_policies/chorus/standard_publication_model>; version="vor"; rel="license", <http://orcid.org/0000-0002-5924-1007>; title="Maria Siopi"; rel="author", <http://orcid.org/0000-0002-8869-1509>; title="Joseph Meletiadis"; rel="author", <http://orcid.org/0000-0001-5087-4003>; title="Aristides Dokoumetzidis"; rel="author"
<http://dx.doi.org/10.1111/jphp.12275>; rel="canonical", <https://api.wiley.com/onlinelibrary/tdm/v1/articles/10.1111%2Fjphp.12275>; version="vor"; rel="item", <https://api.wiley.com/onlinelibrary/tdm/v1/articles/10.1111%2Fjphp.12275>; version="vor"; type="application/pdf"; rel="item", <http://academic.oup.com/jpp/article-pdf/66/10/1421/36221699/jphp12275.pdf>; version="vor"; type="application/pdf"; rel="item", <http://academic.oup.com/jpp/article-pdf/66/10/1421/36221699/jphp12275.pdf>; version="vor"; rel="item", <https://academic.oup.com/journals/pages/open_access/funder_policies/chorus/standard_publication_model>; version="vor"; rel="license", <http://orcid.org/0000-0002-4885-3745>; title="Silvia Romano-Moreno"; rel="author
<http://dx.doi.org/10.1211/jpp.59.8.0004>; rel="canonical", <https://api.wiley.com/onlinelibrary/tdm/v1/articles/10.1211%2Fjpp.59.8.0004>; version="vor"; rel="item", <http://academic.oup.com/jpp/article-pdf/59/8/1077/36739097/jpp.59.8.0004.pdf>; version="vor"; type="application/pdf"; rel="item", <http://academic.oup.com/jpp/article-pdf/59/8/1077/36739097/jpp.59.8.0004.pdf>; version="vor"; rel="item", <https://academic.oup.com/journals/pages/open_access/funder_policies/chorus/standard_publication_model>; version="vor"; rel="license"
